# Blood pressure, arterial stiffness, and cardiovascular risk profiles in 8–12-year-old children following preeclampsia (FINNCARE-study)

**DOI:** 10.1097/HJH.0000000000003485

**Published:** 2023-06-19

**Authors:** Michelle A.-K. Renlund, Tiina J. Jääskeläinen, Anni S.E. Kivelä, Seppo T. Heinonen, Hannele M. Laivuori, Taisto A. Sarkola

**Affiliations:** a Children's Hospital, University of Helsinki and Helsinki University Hospital, Helsinki, Finland; b Minerva Foundation Institute for Medical Research, Helsinki, Finland; c Medical and Clinical Genetics, University of Helsinki and Helsinki University Hospital, Helsinki, Finland; d Department of Food and Nutrition, University of Helsinki, Helsinki, Finland; e Department of Obstetrics and Gynecology, Helsinki University Hospital, Helsinki, Finland; f Department of Obstetrics and Gynecology, Tampere University Hospital and Tampere University, Faculty of Medicine and Health Technology, Tampere Center for Child, Adolescent, and Maternal Health Research, Tampere, Finland

**Keywords:** adiposity, arterial stiffness, blood pressure, cardiovascular disease, preeclampsia

## Abstract

The aim was to study if children following preeclampsia (PE) develop alterations in blood pressure (BP) and arterial stiffness already early in life, and how this is associated with gestational, perinatal and child cardiovascular risk profiles.

**Methods:**

One hundred eighty-two PE (46 early-onset with diagnosis before 34 gestational weeks, and 136 late-onset) and 85 non-PE children were assessed 8–12 years from delivery. Office and 24-h ambulatory BP, body composition, anthropometrics, lipids, glucose, inflammatory markers, and tonometry-derived pulse wave velocity (PWV) and central BPs were assessed.

**Results:**

Office BP, central BPs, 24-h systolic BP (SBP) and pulse pressure (PP) were higher in PE compared with non-PE. Early-onset PE children had the highest SBP, SBP-loads, and PP. SBP nondipping during night-time was common among PE. The higher child 24-h mean SBP among PE was explained by maternal SBP at first antenatal visit and prematurity (birth weight or gestational weeks), but child 24-h mean PP remained related with PE and child adiposity after adjustments. Central and peripheral PWVs were elevated in late-onset PE subgroup only and attributed to child age and anthropometrics, child and maternal office SBP at follow-up, but relations with maternal antenatal SBPs and prematurity were not found. There were no differences in body anthropometrics, composition, or blood parameters.

**Conclusions:**

PE children develop an adverse BP profile and arterial stiffness early in life. PE-related BP is related with maternal gestational BP and prematurity, whereas arterial stiffness is determined by child characteristics at follow-up. The alterations in BP are pronounced in early-onset PE.

Clinical Trial Registration information: https://clinicaltrials.gov/ct2/show/NCT04676295

ClinicalTrials.gov Identifier: NCT04676295

## INTRODUCTION

Preeclampsia (PE) is a hypertensive disorder of pregnancy (HDP) with gestational hypertension and new-onset proteinuria and/or maternal organ dysfunction and/or uteroplacental dysfunction at or after 20 weeks of gestation [1]. PE is later related with ischemic heart disease, cerebrovascular disease, and premature cardiovascular disease (CVD) mortality [2]. Studies also show that PE children have an increased CVD risk profile with elevated blood pressure (BP) and increased body mass index (BMI) in childhood and young adulthood [3]. This is later followed by early development of hypertension and stroke [4]. Systolic BP (SBP) and pulse pressure (PP) are major determinants of cardiovascular morbidity and mortality world-wide [5,6].

PE and other HDPs are related with preterm birth and with important perinatal mortality and morbidity [7]. Preterm birth is associated with elevated BPs in adulthood [8], and adolescents born preterm show risk factors for cardiometabolic disease [9]. Adults born at very low birth weight also show elevated BPs with PE increasing the risk [10]. Early-onset PE offspring show higher BPs during early teenage years [11]. There is currently, however, a lack of studies addressing role of maternal gestational PE factors, PP and arterial stiffness in younger children following PE [12,13]. Current guidelines highlight importance of family and perinatal history when evaluating elevated BPs in children [14].

We hypothesized that PE children develop alterations in BPs early in life and that this is reflected in PP, arterial stiffness, and the cardiovascular risk profile overall. Our aim was to prospectively compare children 8–12 years following PE with age matched children without PE or HDP. Our maternal, gestational, and perinatal data set is comprehensive, and we also assessed offspring body anthropometrics and composition, lipids, glucose, and inflammatory markers.

## METHODS

### Study design, sample and setting

The registered FINNCARE study includes both a cross-sectional cohort study design performed at recruitment as well as the randomization of PE families into a behavioral lifestyle 12-month intervention (NCT04676295) [15]. This article reports data from baseline and assesses CVD risk and CVD progression at 8–12 years from delivery. Briefly, FINNCARE study families living in the Hospital district of Helsinki and Uusimaa were recruited randomly from The Finnish Genetics of Preeclampsia Consortium (FINNPEC) multicenter study cohort [16]. Between 2008 and 2011, 1450 nulli- or multiparous women with PE and 1065 without PE (non-PE) were prospectively recruited together with their partners and newborns. PE was defined as hypertension and proteinuria occurring after 20 gestational weeks [SBP ≥ 140 mmHg and/or diastolic BP (DBP) ≥ 90 mmHg, and urinary excretion of ≥ 0.3 g protein in a 24-h specimen, or 0.3 g/l, or two ≥1 + readings on dipstick]. Maternal chronic hypertension was defined as SBP ≥140 mmHg and/or DBP ≥90 mmHg detected before 20 gestational weeks or medication for hypertension before the index pregnancy.

In total, 182 PE and 85 non-PE children consented to participate in the FINNCARE study. Exclusion criteria included ongoing maternal pregnancy or lactation, multiple pregnancy and inability to communicate in Finnish. For non-PE, exclusion criteria also included PE, gestational hypertension or chronic hypertension, gestational diabetes and/or diabetes during or following index pregnancy. To address potential recruitment bias, we compared backgrounds of participating and nonparticipating PE mothers from the Hospital district of Helsinki and Uusimaa FINNPEC cohort with no major differences observed (Table 1, Supplemental Digital Content 1).

Study visits were arranged between June 2019 and June 2022 with 2:1 PE and non-PE families scheduled per visit in a tertiary care setting at the Clinical Trial Unit at Children's Hospital, Helsinki, Finland. Personnel performing study visits, collection and analyses of raw data were blinded to participant PE status. Participation was confirmed with a signed informed consent. The study protocol has approval by the Ethics Committee of the Hospital District of Helsinki and Uusimaa (HUS/3347/2018).

### Blood pressures

Office BP was measured three times from the nondominant arm using appropriately sized cuffs following a one-hour rest and with the subject sitting upright [14]. The BP monitor device Omron HBP-1300 was changed mid-study to Omron HBP-1320 (see Supplementary Methods, Supplemental Digital Content 1). SBP, DBP and heart rate (HR) are reported as mean of last two measurements. PP was calculated as the difference between SBP and DBP. SBP and DBP *z*-scores were generated for age, height and sex [14].

Ambulatory blood pressures (ABP) were assessed during 24 h from the nondominant arm with an oscillometric Schiller BR-102 plus device every 30-min during daytime and at 1-h intervals during night-time in accordance with diary information [17]. Eighteen night-time and 16 daytime child registrations were discarded due to outliers or <65% valid measurements during monitoring [17]. Office and ABP devices provided similar results (see Supplementary Methods, Supplemental Digital Content 1).

We calculated BP z-scores for height and age [18], standard deviations (SDs), coefficients of variations (CVs), weighted 24-h SDs separately for daytime and night-time, and BP dip and loads [17]. We further classified BP dipping in four categories: nondipping (0–10% decrease), normal dipping (10–20% decrease), extreme dipping (>20% decrease), and reversed dipping (>0% increase) [17].

### Anthropometrics

Height and weight were measured with a Seca 285 scale and stadiometer (Seca GmBH & Co., Hamburg, Germany) to the closest 0.1 centimeter and 0.05 kg, respectively. Waist and hip circumferences were measured to the closest 0.1 centimeter. Fat mass, lean body mass (LBM), skeletal muscle mass, and body fat percentage were assessed with bioelectrical impedance (InBody 720; InBody Bldg, Korea). LBM was also calculated [19]. We calculated body surface area (BSA) with Haycock formula, and generated height, BMI and weight *z*-scores for sex and age as well as weight z-score for sex and height [20].

### Pulse wave velocity and pulse wave analysis

Carotid-femoral (CF-PWV) and carotid-radial (CR-PWV) assessments were performed at rest in a supine position in a quiet examination room with mean of two high-quality measurements used in analyses (Complior Analyse; Alam Medical, Saint-Quentin-Fallavier, France). Distances were measured to the nearest 0.1 centimeter, and the carotid-femoral distance was multiplied by 0.8. Central aortic SBP, DBP, and PP were automatically generated by the device from the carotid waveform and calibrating the signal with diastolic and mean office brachial BP.

### Blood work parameters

Blood work parameters from venous overnight fasting blood samples were determined with standard hospital laboratory methods.

### Questionnaire and index pregnancy data

We collected family backgrounds, household annual income, parental smoking and alcohol intake with standard questionnaires. Index pregnancy and perinatal data was obtained from the FINNPEC database [16]. Highest maternal BP during pregnancy was missing 59 PE and nine non-PE women. Birth anthropometrics *z*-scores were generated [21]. Early-onset PE was analyzed separately as diagnosis or delivery before 34^0/7^ gestational weeks. Prematurity was defined as delivery before 37 gestational weeks and small for gestational age (SGA) as birth weight below −2SD. Hemolysis, elevated liver enzymes, and low platelet count (HELLP) syndrome was diagnosed when at least two of the following criteria were met: lactate dehydrogenase ≥235 U/l, alanine aminotransferase ≥70 U/l, aspartate aminotransferase ≥70 U/l, thrombocytes ≤100 E9/l. Placental insufficiency was defined as umbilical artery Doppler pulsatility index (PI) >+2SD or resistance index (RI) >+2SD.

### Data analysis

Data are presented as mean (SD), median (interquartile range), and count (percentage), as appropriate. Normality was checked with histograms, *Q*−*Q* plots, normality tests (Kolmogorov–Smirnov and Shapiro–Wilk) and skewness. Differences between groups were tested using Independent Samples *t*-test or Mann–Whitney *U*-test, and Pearson Chi-square or two-tailed Fisher's exact test for categorical data. In the PE group, analyses were separately made using birth or diagnosis prior to 34^0/7^ gestational weeks for early-onset PE (and late-onset PE) subgroups (Tables 2–5, Supplemental Digital Content 1) with the analysis providing higher statistical significance reported in Results text. Univariate regressions of PE children's 24-h SBP, PP and PWVs are presented in domain groups (Tables 6 and 7, Supplemental Digital Content 1). We explored adjusted 24-h SBP, PP, and CF-PWV mean differences between PE and non-PE separately for each confounder variable using ANCOVA (Table 8, Supplemental Digital Content 1). We then constructed multiple linear regression models to assess the combined effect of confounders on PE related 24-h ambulatory SBP, PP, and CF-PWV (Table 9, Supplemental Digital Content 1). We included models for SBP and PP separately adjusting for prematurity and maternal SBP at first antenatal visit. Child and maternal SBP at follow-up was separately included in CF-PWV models, in addition to child age and prematurity. Normality, homoscedasticity, independence, linearity were assessed, and models were examined for multicollinearity using variance influence factor and collinearity tolerance with a VIF <2.5 and CT >0.3 deemed appropriate. All multiple models were also separately assessed with ANCOVA analyses to showcase adjusted mean differences between PE and non-PE. Statistical analysis was performed using SPSS 27 (IBM, Armonk, New York, USA).

### Independent data access and analysis

T.S. had access to all study data and takes responsibility for its integrity and analysis.

##  RESULTS

### Maternal and perinatal child characteristics

Forty-six PE pregnancies were early-onset based on diagnosis and 25 were delivered before 34^0/7^ gestational weeks with more maternal morbidity and child prematurity and smaller body size for gestational age compared with late-onset PE and non-PE pregnancies (Table 2, Supplemental Digital Content 1). Early-onset PE mothers had more chronic hypertension, higher gestational maximum BPs and more documented placental insufficiency and HELLP syndrome. PE mothers were more often primiparous. There was no difference in age at delivery, smoking during pregnancy, or household income at follow-up (results not shown) between PE and non-PE mothers.

### Child body anthropometrics and composition at 8–12-year follow-up

Sex distribution was similar and PE were slightly older than non-PE at follow-up (Table 3, Supplemental Digital Content 1). There were no major differences in body anthropometrics or weight categories (severely underweight, underweight, overweight, and obese). Age-adjusted height *z*-score was lower in the early-onset PE subgroup [mean difference −0.90 (95% CI −1.46 to −0.34)]. No PE-related difference in BMI *z*-score or body composition parameters (skeletal muscle mass, lean body mass, fat mass, and body fat percentage) were found.

### Blood pressure level and variability in children at 8–12-year follow-up

Office SBP and SBP *z*-score were similarly higher in both early- and late-onset PE compared with non-PE children (mean difference +5.3 mmHg (95% CI 3.2–7.4); Table 1; Table 4, Supplemental Digital Content 1). Central SBP was also similarly higher [mean difference +6.7 mmHg (95% CI 3.7–9.7) for PE and mean difference +9.8 mmHg (95% CI 5.5–14.1) for early-onset PE]. Office DBP and DBP *z*-score were higher in PE children compared with non-PE children [mean difference +1.8 mmHg (0.3–3.4)]. This difference was accentuated in the early-onset PE subgroup with a mean difference of +4.6 mmHg (95% CI 1.9–7.3). Office PP was higher in PE children compared with non-PE children [mean difference +3.2 mmHg (95% CI 1.3–5.1)]. Heart rate at BP assessment was no different.

**TABLE 1:** Blood pressure and PWV

	Non-PE	PE	Early-onset PE	Late-onset PE	Mean difference	Mean difference	Mean difference
			Diagnosis	Diagnosis	(95% CI)	(95% CI)	(95% CI)
			<34^0/7^ weeks	≥34^0/7^ weeks	PE vs. non-PE	Early-onset PE vs. non-PE	Late-onset PE vs. non-PE
Office blood pressure	*N =* *85*	*N =* *182*	*N =* *46*	*N =* *136*			
SBP (mmHg)	109.6 (7.4)	114.9 (9.7)	115.2 (9.4)	114.8 (9.8)	5.3 (3.2–7.4)^§^	5.6 (2.7–8.6)^§^	5.2 (2.9–7.5)^§^
DBP (mmHg)	68.6 (5.8)	70.4 (6.2)	71.2 (6.5)	70.1 (6.1)	1.8 (0.3–3.4)^#^	2.7 (0.5–4.9)^#^	1.5 (−0.1–3.2)
PP (mmHg)	41.0 (6.6)	44.2 (7.7)	43.8 (8.7)	44.4 (7.4)	3.2 (1.3–5.1)^||^	2.7 (0.1–5.4)^#^	3.4 (1.4–5.3)^§^
Central blood pressures	*N =* *79*	*N =* *174*	*N =* *44*	*N =* *130*			
Central SBP (mmHg)	103.0 (8.7)	109.7 (12.2)	112.8 (12.8)	108.6 (11.8)	6.7 (3.7–9.7)^§^	9.8 (5.5–14.1)^§^	5.6 (2.8–8.4)^§^
Central DBP (mmHg)	71.0 (6.1)	71.9 (6.0)	72.3 (6.4)	71.8 (5.9)	0.9 (−0.7–2.5)	1.3 (−1.0–3.6)	0.7 (−0.9–2.4)
Central PP (mmHg)	31.5 (10.0)^∗^	36.6 (15.0)^∗^	38.5 (16.0)^∗^	36.3 (14.0)^∗^	5.0 (2.5–7.5)^§^	7.0 (3.3–11.0)^§^	4.33 (1.67–7.00)^||^
Pulse wave velocity	*N =* *79*	*N =* *174*	*N =* *44*	*N =* *130*			
Carotid-femoral (m/s)	5.12 (0.64)	5.32 (0.81)	5.05 (0.58)	5.41 (0.85)	0.20 (0–0.41)^#^	−0.07 (−0.30–0.17)	0.29 (0.08–0.51)^||^
Carotid-radial (m/s)	7.41 (1.03)	7.84 (1.19)	7.68 (1.32)	7.89 (1.15)	0.43 (0.12–0.73)^||^	0.27 (−0.16–0.70)	0.48 (0.17–0.79)^||^
24-h blood pressure	*N =* *63*	*N =* *144*	*N =* *35*	*N =* *109*			
SBP (mmHg)	119.6 (6.8)	122.5 (8.8)	125.1 (9.7)	121.7 (8.4)	2.9 (0.4–5.3)^#^	5.5 (1.7–9.2)^||^	2.0 (−0.4–4.5)
DBP (mmHg)	71.3 (5.4)	70.4 (5.8)	70.2 (6.3)	70.5 (5.6)	−0.9 (−2.6–0.8)	−1.2 (−3.5–1.3)	−0.8 (−2.6–0.9)
PP (mmHg)	48.4 (5.2)	52.1 (7.6)	55.1 (9.4)	51.1 (6.7)	3.7 (1.9–5.4)^§^	6.7 (3.2–10.1)^§^	2.7 (0.8–4.6)^||^
SBP load (%)^†^	34.8 (20.2)	43.1 (26.2)	53.0 (26.3)	39.9 (25.5)	8.3 (1.7–14.9)^#^	18.2 (7.9–28.5)^§^	5.1 (−1.8–12.1)
DBP load (%)^†^	27.0 (18.1)	25.1 (16.6)	24.5 (17.5)	25.3 (16.3)	−1.9 (−7.0–3.2)	−2.5 (−10.0–5.0)	−1.7 (−7.0–3.6)
SBP nocturnal dip (%)	11.5 (6.9)	11.2 (6.0)	9.5 (6.1)	11.7 (5.9)	−0.3 (−2.2–1.6)	−1.9 (−4.7–0.8)	0.2 (−1.8–2.2)
DBP nocturnal dip (%)	14.8 (9.7)	15.2 (8.3)	15.4 (9.3)	15.1 (8.0)	0.4 (−2.3–3.0)	0.6 (−3.4–4.6)	0.3 (−2.4–3.0)
Daytime	*N =* *71*	*N =* *154*	*N =* *38*	*N =* *116*			
SBP (mmHg)	123.5 (7.6)	126.1 (8.9)	128.2 (9.8)	125.4 (8.5)	2.6 (0.2–5.0)^#^	4.7 (1.1–8.4)^#^	1.9 (−0.5–4.3)
DBP (mmHg)	73.9 (6.4)	73.4 (6.1)	73.4 (6.9)	73.4 (5.8)	−0.5 (−2.3–1.2)	−0.5 (−3.1–2.1)	−0.5 (−2.3–1.3)
PP (mmHg)	49.6 (5.0)	52.7 (7.4)	54.8 (8.7)	52.1 (6.8)	3.1 (1.5–4.8)^§^	5.2 (2.1–8.2)^||^	2.4 (0.7–4.1)^||^
SBP load (%)^†^	35.1 (21.0)	43.4 (27.2)	52.2 (27.7)	40.5 (26.6)	8.3 (1.7–14.8)^#^	17.0 (6.8–27.3)^||^	5.4 (−1.5–12.3)
DBP load (%)^†^	19.0 (28.0)^∗^	17.5 (23.0)^∗^	19.5 (25.0)^∗^	17.0 (23.0)^∗^	−1.0 (−6.0–4.0)	0 (−8.0–6.0)	−1.0 (−6.0–4.0)
night-time	*N =* *69*	*N =* *154*	*N =* *38*	*N =* *116*			
SBP (mmHg)	109.0 (9.1)	112.1 (10.7)	115.7 (11.6)	111.0 (10.2)	3.2 (0.3–6.1)^#^	6.8 (2.7–10.8)^||^	2.0 (−0.9–4.9)
DBP (mmHg)	62.8 (7.6)	62.2 (7.3)	61.5 (8.1)	62.4 (7.0)	−0.6 (1.1 to −2.7)	−1.3 (−4.4–1.8)	−0.4 (−2.6–1.7)
PP (mmHg)	46.0 (7.8)	49.7 (8.7)	53.2 (6.7)	48.6 (8.0)	3.7 (1.3–6.1)^||^	7.1 (3.7–10.6)^§^	2.6 (0.2–4.9)^#^
SBP load (%)^†^	32.9 (27.5)	41.1 (31.0)	53.3 (31.7)	37.2 (29.8)	8.2 (−0.3–16.8)	20.4 (8.7–32.0)^§^	4.2 (4.4–12.9)
DBP load (%)^†^	34.0 (26.0)	35.7 (26.2)	32.3 (26.1)	36.8 (26.3)	1.7 (−5.7–9.2)	−1.7 (−12.2–8.7)	2.8 (−5.0–10.7)
SBP dipping^‡^	*N =* *63*	*N =* *144*	*N =* *35*	*N =* *109*	^#^	^#^	−
Nondipping, *n* (%)	13 (20.6)	48 (33.3)	15 (42.9)	33 (30.3)			
Normal dipping, *n* (%)	37 (58.7)	84 (58.3)	18 (51.4)	66 (60.6)			
Extreme dipping, *n* (%)	7 (11.1)	7 (4.9)	0 (0)	7 (6.4)			
Reversed dipping, *n* (%)	6 (9.5)	5 (3.5)	2 (5.7)	3 (2.8)			
DBP dipping^‡^	*N =* *63*	*N =* *144*	*N =* *35*	*N =* *109*	−	−	−
Nondipping, *n* (%)	10 (15.9)	27 (18.8)	9 (25.7)	18 (16.5)			
Normal dipping, *n* (%)	27 (42.9)	74 (51.4)	15 (42.9)	59 (54.1)			
Extreme dipping, *n* (%)	21 (33.3)	37 (25.7)	10 (28.6)	27 (24.8)			
Reversed dipping, *n* (%)	5 (7.9)	6 (4.2)	1 (2.9)	5 (4.6)			

Data is presented as mean (SD) unless stated otherwise. Independent samples *t*-test for normally distributed numerical data, Mann–Whitney *U*-test for nonnormal distribution and Pearson chi-square or Fischer's exact test for categorical data. CI, confidence interval; DBP, diastolic blood pressure; IQR, interquartile range; PE, preeclampsia; PP, pulse pressure; PWV, pulse wave velocity; SBP, systolic blood pressure.

∗ Median (IQR), median difference (95% CI).

† Proportion of measurements exceeding the height- and gender-specific 95th percentile.

‡ Nondipping: blood pressure decrease of 0% or greater, but less than 10%; normal dipping: blood pressure decrease of 10% or greater, but less than 20%; extreme dipping: blood pressure decrease of 20% or greater; reversed dipping; blood pressure increase during night-time.

§ *P*-value <0.001.

|| *P*-value <0.01.

# *P*-value <0.05.

**FIGURE 1:**
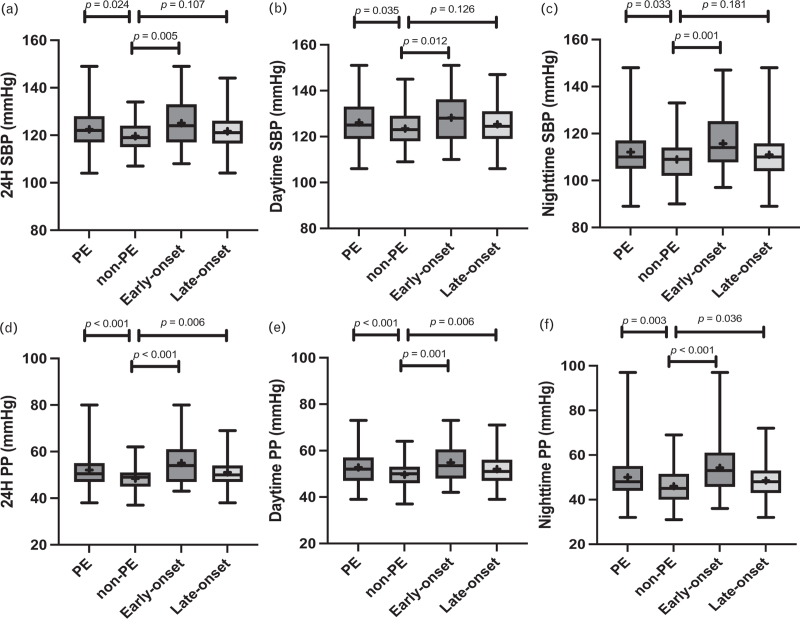
Systolic blood pressure (SBP) and pulse pressure (PP) profiles. (a) 24-h SBP. (b) Daytime SBP. (c) night-time SBP. (d) 24-h PP. (e) Daytime PP. (f) night-time PP. PE indicates preeclamptic group; non-PE, control group; early-onset, preeclampsia diagnosis before 34^0/7^ gestational weeks; late-onset, preeclampsia diagnosis at or after 34^0/7^ gestational weeks. Box-plots with whiskers: mean = the + sign, median = the horizontal line of the box, interquartile range = the box limits, minimum and maximum values = the extent of the whiskers.

Mean 24-h ambulatory SBP and SBP *z*-score were higher in PE compared with non-PE children [mean difference +2.9 mmHg (95% CI 0.4–5.3); Table 1 and Fig. 1; Table 4, Supplemental Digital Content 1]. This difference to non-PE was more pronounced in early-onset compared with late-onset PE [mean difference +5.5 mmHg (95% CI 1.7–9.2) vs. +2.6 mmHg (95% CI 0.2–5.1), respectively]. Mean 24-h ambulatory DBP (or DBP *z*-score) was, however, no different between PE and non-PE. Thus, mean 24-h ambulatory PP was higher in PE (both early-onset and late-onset PE) compared with non-PE [mean difference +3.7 mmHg (1.9–5.4)]. Both daytime and night-time ambulatory SBP (and PP) were similarly higher in PE compared with non-PE. Daytime and night-time DBP were no different between PE and non-PE.

Mean SBP dip during night-time was similar between PE and non-PE groups. There was, however, a higher proportion of nondipping SBP pattern in PE compared with non-PE (33.3 vs. 20.6%, *P* = 0.046; Table 1). This difference was more pronounced among early-onset PE (47.4 vs. 20.6%, *P* = 0.035). Extreme dipping was present in a minority of non-PE (11.1%) and absent among all early-onset PE. Mean DBP dip during night-time or dipping pattern was no different between PE (or PE subgroups) and non-PE. Different measures of 24-h ambulatory SBP or DBP variability (SD, SD weighted for duration of monitoring time, and CV) were no different between PE and non-PE. Minimum night-time SBP was higher among PE (both early and late-onset PE) compared with non-PE (mean 96.5 vs. 90.9 mmHg; *P*-value < 0.001; Table 4, Supplemental Digital Content 1), but there were no differences in maximum SBP during day and night as well as daytime minimum SBP nor in any comparable DBP values.

### Predictors of child blood pressure among preeclampsia children

Univariate regressions were performed to explore predictors of PE children's 24-h ambulatory SBP and PP (Tables 6 and 7, Supplemental Digital Content 1). Gestational time and prematurity, birth weight and SGA, parity (multiparity), maternal SBP at first antenatal visit, and highest SBP during gestation were all associated with ambulatory SBP. There was no association with maternal office SBP at follow-up. Child follow-up weight *z*-score, waist circumference, waist-hip ratio, and BMI *z*-score, as well as office and central systolic BP, but not sex, were also associated with PE children's ambulatory SBP. These associations were overall similar during day and night-time, but associations with child anthropometrics and adiposity measures were consistently significant only during night-time. Prematurity, parity (multiparity), maternal prepregnancy BMI, and highest maternal SBP during gestation were all associated with PE child ambulatory 24-h PP in univariate analyses. In these analyses all child anthropometric and adiposity measures showed strong associations with child ambulatory PP both during daytime and night-time.

As maternal parity was associated with 24-h ambulatory BP and PP among PE children we explored differences in maternal pregnancy and perinatal characteristics data between primiparous PE and multiparous PE mothers (Table 10, Supplemental Digital Content 1). No differences were, however, found. We also found no associations between parity and 24-h ambulatory SBP and PP among non-PE children.

Twenty-four-hour ambulatory SBP mean difference [2.9 mmHg (95% CI 0.4–5.3)] between PE and non-PE children remained significant when separately adjusting for child waist-hip ratio, BMI *z*-score, and weight *z*-score at follow-up, and with maternal parity at index pregnancy (Table 8, Supplemental Digital Content 1). The SBP mean difference was, however, attenuated to nonsignificant levels when separately adjusting for birth weight, birth weight *z*-score, gestational age at delivery, and maternal SBP at first antenatal visit.

Twenty-four-hour PP mean difference [3.7 mmHg (95% CI 1.9–5.4)] between PE and non-PE children remained, however, significant when separately adjusting for maternal SBP at first antenatal visit, maternal parity, maternal prepregnancy BMI, prematurity, gestational age at delivery, child birth weight, and using different child anthropometrics and adiposity measures at follow-up (Table 8, Supplemental Digital Content 1).

The combined effect of different predictors on child 24-h SBP was then assessed with multiple linear regression models (Table 9, Supplemental Digital Content 1) and ANCOVA analyses outlining mean differences between PE and non-PE (Table 2). Child BMI *z*-score at follow-up and parity improved the model. Adjustment with child birth weight further improved the model with all predictors except PE remaining significant. Replacing child birth weight with maternal SBP at first antenatal visit provided similar results. In the final ANCOVA model with all predictors included the adjusted 24-h SBP mean difference between PE and non-PE was 0.2 mmHg (95% CI –2.5–2.9).

**TABLE 2:** Adjusted mean differences for preeclamptic versus nonpreeclamptic children

Outcome	Model	Mean difference (95% CI)	*P*-value
24-h SBP	Unadjusted mean difference	**2.9 (0.4–5.3)**	**0.024**
	Child BMI *z*-score at follow-up	2.9 (0.5–5.3)	**0.020**
	Child BMI *z*-score + parity	3.2 (0.7–5.6)	**0.011**
	Child BMI *z*-score + parity + child birth weight	1.5 (−1.2–4.1)	0.270
	Child BMI *z*-score + parity + maternal first SBP at antenatal visit	2.0 (−0.6–4.6)	0.124
	Child BMI *z*-score + parity + child birth weight + maternal first SBP at antenatal visit	0.2 (−2.5–2.9)	0.890
24-h PP	Unadjusted mean difference	**3.7 (1.9–5.4)**	**<0.001**
	Child BMI *z*-score	3.7 (1.8–5.6)	**<0.001**
	Child BMI *z*-score + parity	4.1 (2.2–6.0)	**<0.001**
	Child BMI *z*-score + parity + child birth weight	2.7 (0.7–4.8)	**0.010**
	Child BMI *z*-score + parity + maternal first SBP at antenatal visit	3.5 (1.5–5.5)	**<0.001**
	Child BMI *z*-score + parity + child birth weight + maternal first SBP at antenatal visit	2.0 (−0.2–4.1)	0.071
CF-PWV	Unadjusted mean difference	**0.20 (0–0.41)**	**0.049**
	Child birth weight	0.32 (0.09–0.55)	**0.006**
	Child birth weight + child age at follow-up	0.26 (0.04–0.48)	**0.022**
	Child birth weight + child age + child office SBP	0.18 (−0.04–0.40)	0.101
	Child birth weight + child age + maternal office SBP	0.20 (−0.02–0.42)	0.078
	Child birth weight + child age + child office SBP + maternal office SBP	0.13 (−0.09–0.35)	0.228

Significant *P*-values (<0.05) bolded. BMI, body mass index; CF-PWV, carotid-femoral pulse wave velocity; CI, confidence interval; PE, preeclampsia; PP, pulse pressure; SBP, systolic blood pressure.

Similar multiple linear regression models (Table 9, Supplemental Digital Content 1) and ANCOVA models (Table 2) were assessed for child 24-h PP. Child birth weight improved the model with all predictors, including PE, statistically significant. Replacing child birth weight with maternal SBP at first antenatal visit provided similar results. In the final ANCOVA model with all predictors included the adjusted 24-h PP mean difference between PE and non-PE was 2.0 mmHg (95% CI –0.2–4.1; *P*-value = 0.071). *R*^2^ values were overall higher for child 24-h PP models compared with 24-h SBP models (*R*^2^ = 0.248 vs. 0.126, respectively).

### Pulse wave velocity in children at 8–12-year follow-up

Carotid-femoral (central aortic) PWV was higher in PE compared with non-PE (mean difference 0.20 m/s (95% CI 0–0.41), Table 1; Table 4 and Figure 1, Supplemental Digital Content 1). This difference was attributed to the late-onset PE subgroup [mean difference 0.29 m/s (95% CI 0.08–0.51)] with no difference among early-onset PE. Similarly, carotid-radial (peripheral) PWV was higher in PE compared with non-PE [mean difference 0.43 m/s (95% CI 0.12–0.73)], and explained by the late-onset PE subgroup [mean difference 0.48 m/s (95% CI 0.17–0.79)].

### Predictors of pulse wave velocity among preeclampsia children

Univariate regressions were performed to explore predictors of PE children's CF-PWV and CR-PWV (Table 7, Supplemental Digital Content 1). Child birth height, birth weight and birth head circumference were all associated with CF-PWV. Child age and anthropometrics at follow-up, as well as child office SBP, DBP, HR, DBP *z*-score at follow-up, and maternal office SBP at follow-up, but not sex, were all associated with CF-PWV. There was no clear association between adiposity measures and CF-PWV. CR-PWV was only associated with SGA, and child weight *z*-score, BMI *z*-score and body fat percentage at follow-up, as well as child office SBP, DBP, HR and DBP z-score at follow-up.

CF-PWV mean difference (0.20 m/s (0–0.41)) between PE and non-PE children remained significant when separately adjusting for child birth weight and office HR at follow-up visit (Table 8, Supplemental Digital Content 1). CF-PWV mean difference between PE and non-PE was attenuated to nonsignificant levels when separately adjusting for the following follow-up variables: child age, body height, body weight, child office SBP and maternal office SBP.

The combined effect of different predictors on child CF-PWV was then assessed with multiple linear regression models (Supplementary Table 9, Supplemental Digital Content 1) and with ANCOVA analyses outlining mean differences (Table 2). Major improvement in the model was observed when adding child age. Adding child SBP at follow-up further improved the model, but at this stage PE was attenuated to a nonsignificant level. A similar result was obtained replacing child office SBP with maternal office SBP at follow-up. The final model including all variables improved R^2^ with all predictors except PE remaining significant. In the final ANCOVA model, the adjusted CF-PWV mean difference between PE and non-PE was 0.13 m/s [95% CI –0.09–0.35; *P*-value = 0.228; *R*^2^ = 0.163).

### Blood work parameters

Fasting blood glucose, insulin, and calculated HOMA-IR, fasting blood lipids (total cholesterol, LDL-cholesterol, HDL-cholesterol, and triglycerides), hs-CRP, creatinine, alanine aminotransferase and uric acid did not differ between PE (early-onset and late-onset PE) and non-PE children (Table 5, Supplemental Digital Content 1).

## DISCUSSION

This study reports higher SBP, DBP, corresponding central BPs, PP, and PWV among 8–12-year-old PE children compared with matched non-PE children, but no major differences in body anthropometrics, body composition or different laboratory parameters of cardiovascular risk. There was a higher proportion of SBP nondipping among PE and SBP and PP-values were pronounced among early-onset PE. Both maternal gestational SBP and child prematurity factors explained PE-related differences in child SBP at follow-up. Child PP differences between PE and non-PE, however, remained significant after adjustments with gestational SBP and prematurity. Child PP was associated with child adiposity at follow-up. Central and peripheral PWVs were higher among PE and related with child age, anthropometrics, and office SBP at follow-up, but not with gestational maternal SBP or child prematurity factors. There was no influence of sex on PE children's BP or PWV. Taken together, these results show adverse BP profiles predicted by maternal gestational BP and child prematurity as well as arterial stiffness predicted by child anthropometrics and BP at follow-up in children following PE. These findings are consistent with an early emerging cardiovascular risk profile in children following PE and consistent with reported cardiovascular morbidity and mortality in this population later during adulthood [4].

Our office and ambulatory SBP and DBP results confirm recent meta-analyses [3]. Our study shows pronounced systolic BPs among early-onset PE children and is consistent with previous preliminary small sample trends and findings in similar age groups [11,12]. Our results on PE-related elevated systolic BPs both during day and night-time are also consistent with earlier results for 9–12-year-old PE or gestational hypertension disorder children [13,22], and for results in young adults born early preterm [8]. Although we show difference in office diastolic BPs we found no difference in ambulatory diastolic BPs between PE and non-PE groups, which is in contrast with previous reports [22]. However, our results further add to the literature by showing a higher proportion of nondipping SBP and absence of extreme dipping SBP patterns in the early-onset PE compared with non-PE. Early-onset PE children also presented with highest SBP values and loads during ambulatory BP monitoring, but we were unable to show a significant difference between PE and non-PE in other ambulatory BP variability parameters. Our results further show that brachial office SBP results are essentially mirrored in central corresponding BPs with a tendency for pronounced SBP differences between PE and non-PE groups. We show that the PE-related SBP was related with both gestational maternal SBP and child prematurity, both mediating the association. Our results further show an independent association between child BMI at follow-up and SBP, but as no consistent associations between BPs and adiposity measures (fat mass or fat percentage) could be found, we conclude that this is likely explained by anthropometrics including lean body mass in our predominantly nonobese study population. Furthermore, our study found no major differences in anthropometric or adiposity measures between PE and non-PE children, although early-onset PE children were slightly shorter. Interestingly, we found an independent positive association between multiparity and child SBP among PE, but were unable to show this to be attributed to maternal or perinatal factors related with child SBP.

To our understanding, PP has not been previously assessed in PE offspring. Due to limited effects of PE on DBP, analyzing PP provided the opportunity to assess more subtle PE-related differences in SBP. Consistent with this, PPs were higher among PE compared with non-PE children. The difference in PP between the groups was relatively strongly influenced by similar maternal and child factors as for SBP. Our analyses show an independent difference in PP between PE and non-PE groups when adjusting for maternal gestational SBP and child prematurity factors. PP was, furthermore, strongly related with not only BMI but also with different adiposity measures at follow-up suggesting a link between BP and adiposity. As PP is like SBP strongly related with cardiovascular disease long-term [5,6], this provides additional evidence linking PE with cardiovascular disease evolving later in adulthood.

Strong relations between maternal BPs and child BPs might be explained by shared genetic, in-utero, or postnatal lifestyle pathways [23]. Women with a genetical predisposition for hypertension have a higher risk of developing PE and PE with severe features [24]. In the present sample, early-onset PE children displayed the most adverse BP profile. Early-onset and late-onset PE are believed to be of different origins with different placental events affecting the outcomes [25]. Several studies have, like our results, found no clear associations between HDP and inflammatory markers, lipids, glucose and insulin [3,13].

Our late-onset PE children displayed higher PWVs consistent with arterial stiffness. Another recent study showed elevated PWVs among 2–10-year-old children, but confined to early-onset PE children [12]. No association between HDPs and peripheral PWV has previously been reported [13]. Our PWV results were, however, not like child BPs related with maternal gestational BPs or child prematurity, but with child age, body anthropometrics and SBP at follow-up during the time of PWV assessment. Furthermore, we were unable to show relations between PWV and different adiposity measures. Taken together, our interpretation is that arterial stiffness, as measured by PWV, is in the PE prepubertal child more influenced by age, body size and BP at the time of measurement rather than gestational or prematurity factors.

Strengths of the study include the prospective design, the large sample size with an age matched control group, comprehensive background information for both index pregnancy and follow-up as well as comprehensive assessment of BP. The study is limited, like most prospective cohort studies, by loss to follow-up in the original FINNPEC cohort, and some selection bias cannot be fully ruled out. Postnatal child growth data was not available.

In conclusion, significantly higher SBP, DBP, corresponding central BPs, PP, and PWV, but no major differences in body anthropometrics, body composition or blood glucose, lipids or inflammation indicating cardiovascular risk are found among PE children at mean 11 years-of-age. PE-related BP is associated with both maternal gestational systolic BP and child prematurity, whereas arterial stiffness is determined by child anthropometrics and BP at follow-up. Child BP changes are pronounced in the early-onset form of PE.

### Perspectives

Children born from PE develop elevated SBP and PP already during prepubertal age. Early timing of PE onset, more severe gestational maternal SBPs and more severe child intrauterine growth restriction and prematurity are all related with increasing SBP in the child. PP is strongly associated also with child adiposity, but overweight, obesity or metabolic abnormalities are not found in prepubertal PE children. Late-onset PE children show body size and BP-related increased arterial stiffness. Shared maternal and child genetic and lifestyle factors likely explain elevated child BP that potentially could be modified with early screening and lifestyle counseling.

## ACKNOWLEDGEMENTS

Study coordinator Maria Finne and study nurse Eija Kortelainen are acknowledged for their excellent coordination of study visits and data collection management.

Sources of funding: The FINNCARE and FINNPEC studies are supported by grants from Emil Aaltonen Foundation, Academy of Finland, Jane and Aatos Erkko Foundation, Sigrid Juselius Foundation, Finnish Foundation for Laboratory Medicine, Finnish Foundation for Pediatric Research, Medical Society of Finland, Medicinska understödsföreningen Liv och Hälsa, Medicinska stiftelsen i Vasa, NovoNordisk Foundation, Dorothea Olivia, Karl Walter and Jarl Walter Perklén Foundation, Päivikki and Sakari Sohlberg Foundation, Juho Vainio Foundation, and Research Funding of the Helsinki-Uusimaa Hospital District.

### Conflicts of interest

There are no conflicts of interest.

## Supplementary Material

Supplemental Digital Content
