# Supplementary material for: Blood pressure, arterial stiffness, and cardiovascular risk profiles in 8–12-year-old children following preeclampsia (FINNCARE-study)
Source: J Hypertens. 2023 Jun 19;41(9):1429–37. doi: 10.1097/HJH.0000000000003485 (PMC10399950; doi:10.1097/HJH.0000000000003485)
Supplement: Supplemental Digital Content [file jhype-41-1429-s001.pdf]

Online Supplemental Digital Content 1

Blood pressure, arterial stiffness, and cardiovascular risk profiles in 8-12-year-old children following pre-eclampsia (FINNCARE-study)

Michelle Renlund[a,b], Tiina J Jääskeläinen[c,d], Anni Kivelä[c], Seppo Heinonen[e], Hannele Laivuori[c,f], and Taisto Sarkola[a,b]

[a]Children's Hospital, University of Helsinki and Helsinki University Hospital, Helsinki, Finland
[b]Minerva Foundation Institute for Medical Research, Helsinki, Finland
[c]Medical and Clinical Genetics, University of Helsinki and Helsinki University Hospital, Helsinki, Finland
[d]Department of Food and Nutrition, University of Helsinki, Helsinki, Finland
[e]Department of Obstetrics and Gynecology, Helsinki University Hospital, Helsinki, Finland
[f]Department of Obstetrics and Gynecology, Tampere University Hospital and Tampere University, Faculty of Medicine and Health Technology, Tampere Center for Child, Adolescent, and Maternal Health Research, Tampere, Finland

Address for Correspondence: Michelle Renlund, MD, Children's Hospital, Helsinki University Hospital, Stenbäckinkatu 9, POB 281, FIN-00029, Helsinki, Finland.
E-mail address: michelle.renlund@helsinki.fi

SUPPLEMENTARY METHODS

Power calculations in FINNCARE

Power calculations were in FINNCARE performed to detect 5.5 mmHg reduction in maternal systolic BP with a power of 80% and a 2-sided p-value of 0.05 for the difference between the groups in the RCT-study (PE intervention and PE control). When expecting 70% participation rate and allowing for a 20% loss to follow-up or missing data, the aim was to recruit 296 PE families to randomize. Furthermore, the aim was to recruit 100 non-PE parallel control families.

Comparison of Omron HBP-1300 and HBP-1320 devices

The two devices were compared with simultaneous BP assessments in 27 children with SBP mean difference -0.54 mmHg (95% limits of agreement (LoA) -2.40 to +1.33), DBP mean difference -3.92 mmHg (95% LoA -5.46 to -2.38; similar systematic bias over the 70-90 mmHg measurement range), and heart rate (HR) mean difference +0.06 beats per minute (95% LoA -0.60 to +0.71). 3.92 mmHg was then reduced from DBP measurements obtained with Omron HBP-1300.

Comparison of Schiller BR-102 plus and HBP Omron HBP-1320 devices

We compared office (Omron HBP-1320) and Schiller BR-102 plus ABP devices with simultaneous BP assessments in 36 children and 45 mothers with correlations ($r=0.78$, $p<0.0001$) and mean difference -3.2 mmHg (95% LoA -23.5-17.1) for SBP, and correlations ($r=0.79$, $p<0.0001$) and mean difference 2.9 mmHg (95% LoA -11.2-17.0) for DBP.

Precision of pulse wave velocity analysis

The intra-observer, inter-observer and test-retest CVs for CF-PWV were 0.98%, 6.61%, and 4.48%, respectively. The intra-observer, inter-observer and test-retest CVs for CR-PWV were 1.87%, 5.30%, and 5.20%, respectively.

SUPPLEMENTARY FIGURES

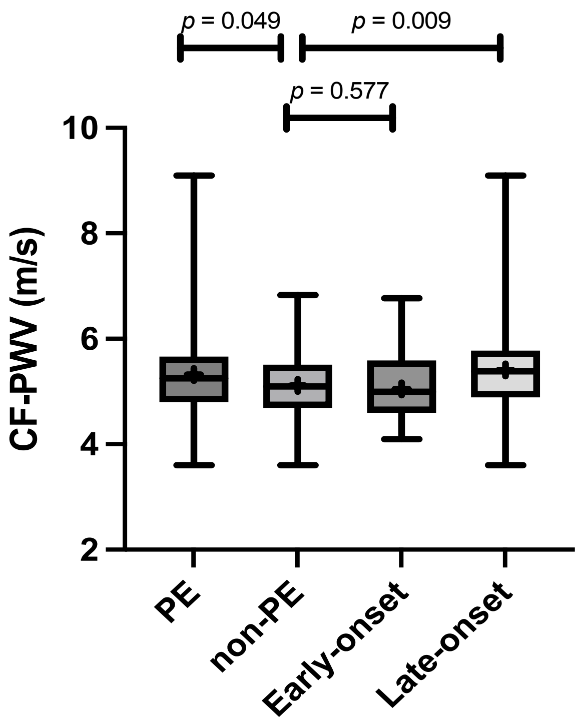

Supplementary Figure 1. Carotid-femoral pulse wave velocity (PWV) profile. PE indicates pre-eclamptic group; non-PE, control group; early-onset, pre-eclampsia diagnosis before 34 $^{0/7}$ gestational weeks; late-onset, pre-eclampsia diagnosis at or after 34 $^{0/7}$ gestational weeks or after. Box-plots with whiskers: mean = the + sign, median = the horizontal line of the box, interquartile range = the box limits, minimum and maximum values = the extent of the whiskers.

SUPPLEMENTARY TABLES

**Supplementary Table 1. FINNCARE study's PE mothers versus non-participant FINNPEC PE mothers**	**Participant PE**	**Non-participant PE**	**Mean difference (95% CI) participant PE versus non-participant PE**	**P-value**
**Child characteristics**	*N = 192*	*N = 118*		
Premature, n (%)	62 (32.3)	35 (29.7)	-	0.628
Gestational weeks	38.0 (4)*	38.0 (3)*	0.00 (-1.0 - 0.00)	0.639
SGA, n (%)	33 (17.2)	21 (17.8)	-	0.891
**Maternal characteristics**				
Primiparous, n (%)	147 (76.6)	82 (69.5)	-	0.169
Pre-pregnancy BMI (kg/m^2)	23.5 (5.1)*	24.6 (8.0)*	-1.0 (-2.0 - -0.1)*	**0.034**
Maternal age at delivery (years)	31.1 (4.6)	31.1 (5.7)	0.03 (-1.2 - 1.3)	0.958
Highest SBP during pregnancy (mmHg)	165.1 (16.3)	165.6 (18.4)	-0.47 (-4.4 - 3.5)	0.816
Highest DBP during pregnancy (mmHg)	109.4 (8.4)	109.7 (9.1)	-0.29 (-2.3 - 1.7)	0.774
Early-onset (diagnosis), n (%)	45 (23.8)	29 (24.6)	-	0.194
Early-onset (birth), n (%)	26 (13.8)	13 (11.0%)	-	0.483
Chronic hypertension, n (%)†	23 (12.0)	21 (17.9)	-	0.145
HELLP syndrome, n (%)	13 (6.8)	2 (1.7)	-	**0.043**
Eclampsia, n (%)	1 (0.5)	0 (0)	-	0.432
Placental insufficiency, n (%)‡	14 (7.3)	10 (8.5)	-	0.705

Data is presented as mean (SD) unless stated otherwise. Independent Samples t Test for normally distributed numerical data, Mann-Whitney U Test for non-normal distribution and Pearson Chi-Square test or Fisher's Exact Test for categorical data. PE indicates pre-eclampsia; BMI, body mass index; SD, standard deviation; IQR, interquartile range; CI, confidence interval; SGA, small for gestational age (birth weight <-2SD); SBP, systolic blood pressure; DBP, diastolic blood pressure; HELLP syndrome, hemolysis, thrombocytopenia and elevated liver enzymes; premature, birth <37+0 gestational weeks; early-onset, pre-eclampsia diagnosis or birth before 34$^{0/7}$ gestational weeks.

*median (IQR), median difference (95% CI)

†SBP ≥ 140 mmHg and/or DBP ≥ 90 mmHg detected before 20 gestational weeks

‡Pulsatility index > +2SD or resistance index > +2SD in umbilical arterial Doppler assessment

Supplementary Table 2. Maternal and perinatal data	non-PE	PE	Early-onset PE *Diagnosis* *<34^0/7 weeks*	Early-onset PE *Birth* *<34^0/7 weeks*	Late-onset PE *Diagnosis* *≥34^0/7 weeks*	Late-onset PE *Birth* *≥34^0/7 weeks*	Mean difference (95% CI) PE versus non-PE
**Child characteristics**	*N = 85*	*N = 182*	*N = 46*	*N = 25*	*N = 136*	*N = 157*	
Premature, n (%)	4 (4.7)	60 (33.0)	42 (91.3)	25 (100.0)	18 (13.2)	35 (22.3)	§
Gestational weeks	40.6 (2.4)*	37.9 (3.6)*	32.9 (3.6)	30.5 (2.9)	38.6 (1.6)	38.3 (1.8)	-2.6 (-3.0- -2.0)§
SGA, n (%)	0 (0)	32 (17.6)	15 (32.6)	12 (48.0)	17 (12.5)	20 (12.7)	§
Birth height (cm)	51.0 (3.0)*	48.0 (4.0)*	41.9 (5.5)	38.4 (4.7)	48.6 (2.3)	48.3 (2.5)	-3.0 (-3.0- -2.0)§
Birth height z-score	-0.04 (0.98)	-0.55 (1.31)	-1.34 (1.53)*	-1.70 (1.42)*	-0.52 (1.50)*	-0.54 (1.53)*	-0.51 (-0.82- -0.20)‖
Birth weight (g)	3614 (54)	2769 (842)	1844 (811)	1271 (445)	3082 (584)	3008 (612)	-845 (-1007- -683)§
Birth weight z-score	0.05 (0.90)	-0.75 (1.27)	-1.18 (1.18)	-1.52 (1.11)	-0.61 (1.27)	-0.63 (1.25)	-0.80 (-1.07- -0.53)§
Head circumference (cm)	35.0 (2.3)*	34.0 (2.5)*	29.5 (4.1)	26.9 (3.6)	34.3 (1.7)	34.1 (1.8)	-1.5 (-2.0- -1.0)§
Head circumference z-score	0.21 (1.01)	-0.18 (1.30)	-0.70 (1.36)*	-0.96 (1.45)*	-0.01 (1.52)*	-0.12 (1.48)*	-0.39 (-0.68- -0.10)‖
**Maternal characteristics**							
Primiparous, n (%)	52 (61.2)	139 (76.4)	33 (71.7)	20 (80.0)	106 (77.9)	119 (75.8)	#
Pre-pregnancy BMI (kg/m^2)	22.5 (4.6)*	23.6 (5.1)*	24.6 (7.4)*	22.5 (5.5)*	23.3 (5.1)*	23.7 (5.5)*	1.1 (0.3-2.0)#
Maternal age at delivery (years)	32.0 (5.3)	31.2 (4.7)	31.5 (4.6)	30.7 (4.6)	31.1 (4.7)	31.3 (4.7)	-0.8 (-2.0-0.5)
PE in previous pregnancy, n (%)	0 (0)	16 (8.8)	4 (8.7)	1 (4.0)	12 (8.8)	15 (9.6)	**Exclusion criteria (control)**
Highest SBP during pregnancy (mmHg)	127.4 (10.4)	173.3 (16.2)	183.2 (15.2)	183.2 (14.2)	170.2 (15.3)	171.7 (16.0)	45.9 (42.2-49.6)§
Highest DBP during pregnancy (mmHg)	81.2 (6.7)	109.3 (8.6)	113.2 (7.3)	113.7 (6.9)	108.1 (8.7)	108.6 (8.7)	28.1 (26.0-30.3)§
Smoking before pregnancy, n (%)	20 (23.5)	38 (21.1)	8 (18.2)	2 (8.7)	30 (22.1)	36 (22.9)	-
Smoking during pregnancy, n (%)	2 (2.4)	9 (5.0)	4 (9.1)	0 (0)	5 (3.7)	9 (5.7)	-
Chronic hypertension, n (%)†	0 (0)	23 (12.6)	11 (23.9)	8 (32.0)	12 (8.8)	15 (9.6)	**Exclusion criteria (control)**
HELLP syndrome, n (%)	0 (0)	14 (7.7)	8 (17.4)	7 (28.0)	6 (4.4)	7 (4.5)	**Exclusion criteria (control)**
Eclampsia, n (%)	0 (0)	1 (0.5)	0 (0.0)	0 (0)	1 (0.7)	1 (0.6)	**Exclusion criteria (control)**
Placental insufficiency, n (%)‡	0 (0)	14 (7.7)	10 (21.7)	9 (36.0)	4 (2.9)	5 (3.2)	‖

Data is presented as mean (SD) unless stated otherwise. Independent Samples t Test for normally distributed numerical data, Mann-Whitney U Test for non-normal distribution and Pearson Chi-Square test or Fisher's Exact Test for categorical data. PE indicates pre-eclampsia; BMI, body mass index; SD, standard deviation; IQR, interquartile range; CI, confidence interval; SGA, small for gestational age (birth weight <-2SD); SBP, systolic blood pressure; DBP, diastolic blood pressure; HELLP syndrome, hemolysis, thrombocytopenia and elevated liver enzymes; premature, birth <37+0 gestational weeks.

*Median (IQR), median difference (95% CI)

†Systolic blood pressure ≥ 140 mmHg and/or diastolic blood pressure ≥ 90 mmHg detected before 20 weeks of gestation

‡Pulsatility index > +2SD or resistance index > +2SD in umbilical arterial Doppler assessment

§p-value <0.001, ||p -value <0.01, #p-value <0.05

Supplementary Table 3. Child characteristics at follow-up	non-PE	PE	Early-onset *Diagnosis* *<34$^{0/7}$ weeks*	Early-onset *Birth* *<34$^{0/7}$ weeks*	Late-onset *Diagnosis* *≥34$^{0/7}$ weeks*	Late-onset *Birth* *≥34$^{0/7}$ weeks*	P-value PE vs non-PE	Mean difference (95% CI)	P-value Early (dg) vs non-PE	Mean difference (95% CI)	P-value Early (birth) vs non-PE	Mean difference (95% CI)	P-value Late (dg) vs non-PE	Mean difference (95% CI)	P-value Late (birth) vs non-PE	Mean difference (95% CI)
**Characteristics & anthropometrics**	*N = 85*	*N = 182*	*N = 46*	*N = 25*	*N = 136*	*N = 157*										
Age (y)	11.2 (1.0)	11.6 (1.1)	11.6 (1.2)	11.8 (1.1)	11.6 (1.1)	11.6 (1.1)	**0.004**	0.4 (0.1-0.7)	**0.035**	0.4 (0-0.8)	**0.020**	0.6 (0.1-1.0)	**0.006**	0.4 (0.1-0.7)	**0.008**	0.4 (0.1-0.7)
Girls (n, %)	42 (49.4)	99 (54.4)	25 (54.3)	14 (56.0)	74 (54.4)	85 (54.1)	0.447	-	0.590	-	0.562	-	0.469	-	0.482	-
Body height (cm)	149.3 (8.4)	151.2 (10.1)	148.7 (9.1)	146.4 (9.0)	152.0 (10.3)	152.0 (10.0)	0.132	1.9 (-0.6-4.4)	0.713	-0.6 (-3.7-2.5)	0.141	-2.9 (-6.7-1.0)	**0.040**	2.7 (0.1-5.4)	**0.039**	2.7 (0.1-5.2)
Body weight (kg)	39.4 (10.4)*	40.3 (17.1)*	41.2 (18.5)*	36.3 (18.4)*	40.1 (16.0)*	40.9 (16.8)*	0.427	1.0 (-1.6-3.5)*	0.477	1.2 (-2.5-5.5)*	0.535	-1.5 (-5.4-3.1)*	0.490	0.9 (-1.7-3.5)*	0.287	1.4 (-1.2-4.0)*
BMI (kg/m^2)	17.8 (3.1)*	17.6 (4.1)*	18.6 (4.7)*	17.2 (4.4)*	17.5 (4.0)*	17.6 (4.1)*	0.993	-0.01 (-0.71-0.70)*	0.215	0.64 (-0.39-1.82)*	0.828	0.11 (-1.11-1.48)*	0.571	-0.19 (-0.93-0.50)*	0.946	-0.03 (-0.75-0.70)*
Body surface area (m^2)†	1.30 (0.23)	1.33 (0.22)	1.33 (0.24)	1.28 (0.22)	1.33 (0.22)	1.34 (0.22)	0.228	0.03 (-0.02-0.08)	0.438	0.03 (-0.05-0.11)	0.652	-0.02 (-0.11-0.07)	0.255	0.03 (-0.02-0.08)	0.141	0.04 (-0.01-0.09)
Height z-score	0.15 (0.89)	0.05 (1.10)	-0.30 (1.18)	-0.75 (1.28)	0.17 (1.06)	0.18 (1.02)	0.450	-0.10 (-0.37-0.17)	**0.015**	-0.45 (-0.81- -0.09)	**0.002**	-0.90 (-1.46- -0.34)	0.918	0.01 (-0.26-0.29)	0.858	0.02 (-0.24-0.28)
Weight z-score (height)	-0.13 (0.95)	-0.22 (1.10)	0.16 (0.97)	0.08 (0.96)	-0.35 (1.11)	-0.27 (1.11)	0.502	-0.09 (-0.36-0.18)	0.102	0.29 (-0.06-0.63)	0.337	0.21 (-0.22-0.64)	0.129	-0.22 (-0.51-0.06)	0.324	-0.14 (-0.42-0.14)
Weight z-score (age)	0.06 (0.96)	-0.06 (1.06)	-0.04 (1.21)	-0.43 (1.23)	-0.06 (1.01)	0.00 (1.02)	0.410	-0.11 (-0.38-0.15)	0.652	-0.09 (-0.51-0.32)	0.078	-0.49 (-1.03-0.06)	0.391	-0.12 (-0.39-0.15)	0.700	-0.05 (-0.32-0.21)
BMI z-score	-0.02 (0.97)	-0.13 (1.08)	0.14 (1.05)	-0.07 (1.02)	-0.22 (1.08)	-0.14 (1.10)	0.443	-0.11 (-0.38-0.17)	0.381	0.16 (-0.20-0.52)	0.833	-0.05 (-0.49-0.39)	0.175	-0.20 (-0.48-0.09)	0.417	-0.12 (-0.39-0.16)
Waist circumference (cm)	63.0 (8.9)*	64.1 (11.0)*	67.0 (12.2)*	64.2 (12.8)*	63.2 (10.5)*	64.0 (10.8)*	0.331	1.0 (-1.0-3.0)*	0.109	2.7 (-0.5-5.9)*	0.402	1.5 (-2.2-5.5)*	0.605	0.5 (-1.5-2.5)*	0.383	1.0 (-1.1-3.0)*
Waist-hip ratio (no unit)	0.82 (0.05)	0.83 (0.06)	0.85 (0.07)	0.85 (0.06)	0.82 (0.06)	0.83 (0.06)	0.582	0 (-0.01-0.02)	**0.038**	0.02 (0-0.05)	**0.043**	0.02 (0-0.05)	0.737	0 (-0.02-0.01)	0.899	0 (-0.01-0.02)
**Body weight categories‡**																
Severely underweight, n (%)	1 (1.2)	5 (2.7)	0 (0)	0 (0)	5 (3.7)	5 (3.2)	0.668	-	1.000	-	1.000	-	0.410	-	0.668	-
Underweight, n (%)	2 (2.4)	6 (3.3)	0 (0)	0 (0)	6 (4.4)	6 (3.8)	1.000	-	0.541	-	1.000	-	0.714	-	0.716	-
Overweight, n (%)	9 (10.6)	17 (9.3)	6 (13.0)	3 (12.0)	11 (8.1)	14 (8.9)	0.749	-	0.674	-	1.000	-	0.529	-	0.672	-
Obese, n (%)	3 (3.5)	8 (4.4)	3 (6.5)	1 (4.0)	5 (3.7)	7 (4.5)	1.000	-	0.423	-	1.000	-	1.000	-	1.000	-
**Body composition**																
Skeletal muscle mass (kg)	17.2 (3.4)	17.9 (4.3)	17.4 (3.9)	16.4 (3.6)	18.0 (4.4)	18.1 (4.3)	0.162	0.7 (-0.3-1.6)	0.730	0.2 (-1.1-1.5)	0.304	-0.8 (-2.3-0.7)	0.115	0.8 (-0.2-1.9)	0.070	0.9 (-0.1-1.9)
Lean mass bioimpedance (kg)	32.6 (5.7)	33.7 (7.2)	32.9 (6.6)	31.2 (6.1)	33.9 (7.4)	34.1 (7.3)	0.175	1.1 (-0.5-2.7)	0.737	0.4 (-1.8-2.6)	0.292	-1.4 (-4.0-1.2)	0.125	1.4 (-0.4-3.1)	0.076	1.5 (-0.2-3.2)
Lean mass calculated (kg)	29.5 (5.8)	30.5 (7.2)	30.3 (7.1)	28.7 (7.0)	30.6 (7.2)	30.8 (7.2)	0.231	1.0 (-0.6-2.6)	0.534	0.8 (-1.7-3.2)	0.562	-0.8 (-3.5-1.9)	0.228	1.1 (-0.7-2.8)	0.135	1.3 (-0.4-2.9)
Fat mass (kg)	6.3 (5.7)*	6.6 (7.1)*	7.5 (9.1)*	5.8 (8.4)*	6.6 (5.6)*	6.8 (6.5)*	0.914	0.1 (-1.0-1.1)*	0.291	0.9 (-0.7-2.9)*	0.839	0.2 (-1.5-2.3)*	0.734	-0.2 (-1.3-0.9)*	0.947	0 (-1.0-1.1)*
Body fat percentage (%)	19.0 (8.3)	18.9 (8.9)	21.2 (9.6)	20.7 (9.7)	18.1 (8.5)	18.6 (8.7)	0.920	-0.1 (-2.4-2.1)	0.170	2.2 (-1.0-5.4)	0.391	1.7 (-2.2-5.6)	0.435	-0.9 (-3.2-1.4)	0.727	-0.4 (-2.7-1.9)

Data is presented as mean (SD) unless stated otherwise, significant p-values (<0.05) bolded. Independent Samples t Test for normally distributed numerical data, Mann-Whitney U Test for non-normal distribution and Pearson Chi-Square test or Fisher's Exact Test for categorical data. PE indicates pre-eclampsia; BMI, body mass index; SD, standard deviation; IQR, interquartile range; CI, confidence interval; dg; diagnosis.

*Median (IQR), median difference (95% CI)

†Calculated with Haycock's formula

‡Severely underweight: girls < 1.3 percentile, boys < 1 percentile; underweight: girls 1.3-5 percentile, boys 1-3.3 percentile; overweight: girls 87.8-98.2 percentile, boys 78.2-95.6 percentile; obese: girls > 98.2 percentile, boys > 95.6 percentile

Supplementary Table 4. Blood pressure and PWV	non-PE	PE	Early-onset *Diagnosis* $<34^{0/7}$ *weeks*	Early-onset *Birth* $<34^{0/7}$ *weeks*	Late-onset *Diagnosis* $\geq 34^{0/7}$ *weeks*	Late-onset *Birth* $\geq 34^{0/7}$ *weeks*	P-value PE vs non-PE	Mean difference (95% CI)	P-value Early (dg) vs non-PE	Mean difference (95% CI)	P-value Early (birth) vs non-PE	Mean difference (95% CI)	P-value Late (dg) vs non-PE	Mean difference (95% CI)	P-value Late (birth) vs non-PE	Mean difference (95% CI)
**Office blood pressure**	*N = 85*	*N = 182*	*N = 46*	*N = 25*	*N = 136*	*N = 157*										
SBP (mmHg)	109.6 (7.4)	114.9 (9.7)	115.2 (9.4)	117.1 (9.3)	114.8 (9.8)	114.6 (9.7)	**<0.001**	5.3 (3.2-7.4)	**<0.001**	5.6 (2.7-8.6)	**<0.001**	7.5 (3.4-11.6)	**<0.001**	5.2 (2.9-7.5)	**<0.001**	5.0 (2.8-7.2)
DBP (mmHg)	68.6 (5.8)	70.4 (6.2)	71.2 (6.5)	73.2 (6.8)	70.1 (6.1)	69.9 (6.0)	**0.023**	1.8 (0.3-3.4)	**0.018**	2.7 (0.5-4.9)	**0.001**	4.6 (1.9-7.3)	0.064	1.5 (-0.1-3.2)	0.086	1.4 (-0.2-3.0)
PP (mmHg)	41.0 (6.6)	44.2 (7.7)	43.8 (8.7)	44.0 (8.7)	44.4 (7.4)	44.3 (7.6)	**0.001**	3.2 (1.3-5.1)	**0.045**	2.7 (0.1-5.4)	0.075	2.9 (-0.3-6.1)	**<0.001**	3.4 (1.4-5.3)	**0.001**	3.3 (1.3-5.2)
HR (bpm)	76.1 (11.3)	76.4 (10.2)	74.8 (9.7)	77.1 (9.5)	76.9 (10.3)	76.3 (10.3)	0.830	0.3 (-2.5-3.1)	0.533	-1.3 (-5.2-2.7)	0.701	1.0 (-4.1-6.0)	0.585	0.8 (-2.1-3.8)	0.893	0.2 (-2.7-3.1)
SBP z-score	0.63 (0.74)	1.01 (0.81)	1.13 (0.76)	1.37 (0.75)	0.97 (0.82)	0.96 (0.80)	**<0.001**	0.38 (0.18-0.59)	**<0.001**	0.50 (0.23-0.78)	**<0.001**	0.74 (0.40-1.08)	**0.002**	0.34 (0.13-0.56)	**0.002**	0.33 (0.12-0.54)
DBP z-score	0.63 (0.58)	0.80 (0.60)	0.92 (0.64)	1.10 (0.66)	0.76 (0.58)	0.75 (0.58)	**0.027**	0.17 (0.02-0.33)	**0.009**	0.29 (0.07-0.51)	**<0.001**	0.47 (0.20-0.75)	0.100	0.13 (-0.03-0.29)	0.106	0.13 (-0.03-0.28)
**Central blood pressures**	*N = 79*	*N = 174*	*N = 44*	*N = 24*	*N = 130*	*N = 150*										
Central SBP (mmHg)	103.0 (8.7)	109.7 (12.2)	112.8 (12.8)	112.6 (10.8)	108.6 (11.8)	109.2 (12.3)	**<0.001**	6.7 (3.7-9.7)	**<0.001**	9.8 (5.5-14.1)	**<0.001**	9.6 (4.7-14.5)	**<0.001**	5.6 (2.8-8.4)	**<0.001**	6.2 (3.4-9.0)
Central DBP (mmHg)	71.0 (6.1)	71.9 (6.0)	72.3 (6.4)	73.5 (6.8)	71.8 (5.9)	71.7 (5.8)	0.283	0.9 (-0.7-2.5)	0.269	1.3 (-1.0-3.6)	0.097	2.5 (-0.5-5.4)	0.386	0.7 (-0.9-2.4)	0.447	0.6 (-1.0-2.3)
Central PP (mmHg)	31.5 (10.0)*	36.6 (15.0)*	38.5 (16.0)*	36.5 (19.0)*	36.3 (14.0)*	36.6 (14.0)*	**<0.001**	5.0 (2.5-7.5)*	**<0.001**	7.0 (3.3-11.0)*	**0.010**	7.2 (1.9-12.5)	**0.001**	4.3 (1.7-7.0)*	**<0.001**	4.8 (2.2-7.3)*
**Pulse wave velocity**	*N = 79*	*N = 174*	*N = 44*	*N = 24*	*N = 130*	*N = 150*										
Carotid-femoral (m/s)	5.12 (0.64)	5.32 (0.81)	5.05 (0.58)	5.02 (0.67)	5.41 (0.85)	5.37 (0.82)	**0.049**	0.20 (0-0.41)	0.577	-0.07 (-0.30-0.17)	0.528	-0.10 (-0.40-0.20)	**0.009**	0.29 (0.08-0.51)	**0.019**	0.25 (0.04-0.46)
Carotid-radial (m/s)	7.41 (1.03)	7.84 (1.19)	7.68 (1.32)	7.76 (1.40)	7.89 (1.15)	7.85 (1.16)	**0.006**	0.43 (0.12-0.73)	0.219	0.27 (-0.16-0.70)	0.187	0.35 (-0.17-0.88)	**0.003**	0.48 (0.17-0.79)	**0.005**	0.44 (0.13-0.75)
**24-hour blood pressure**	*N = 63*	*N = 144*	*N = 35*	*N = 19*	*N = 109*	*N = 125*										
SBP (mmHg)	119.6 (6.8)	122.5 (8.8)	125.1 (9.7)	124.0 (9.7)	121.7 (8.4)	122.3 (8.7)	**0.024**	2.9 (0.4-5.3)	**0.005**	5.5 (1.7-9.2)	0.083	4.3 (-0.6-9.2)	0.107	2.0 (-0.4-4.5)	**0.038**	2.6 (0.2-5.1)
DBP (mmHg)	71.3 (5.4)	70.4 (5.8)	70.2 (6.3)	69.7 (6.3)	70.5 (5.6)	70.5 (5.7)	0.284	-0.9 (-2.6-0.8)	0.345	-1.2 (-3.5-1.3)	0.285	-1.6 (-4.5-1.3)	0.338	-0.8 (-2.6-0.9)	0.347	-0.8 (-2.5-0.9)
PP (mmHg)	48.4 (5.2)	52.1 (7.6)	55.1 (9.4)	54.1 (9.7)	51.1 (6.7)	51.8 (7.2)	**<0.001**	3.7 (1.9-5.4)	**<0.001**	6.7 (3.2-10.1)	**0.023**	5.7 (0.9-10.5)	**0.006**	2.7 (0.8-4.6)	**<0.001**	3.3 (1.5-5.2)
HR (bpm)	81.6 (7.2)	80.9 (7.7)	81.7 (7.8)	83.9 (7.2)	80.6 (7.6)	80.4 (7.7)	0.534	-0.7 (1.1- -3.0)	0.905	0.2 (-2.9-3.3)	0.215	2.3 (-1.4-6.1)	0.400	-1.0 (-3.3-1.3)	0.313	-1.2 (-3.5-1.1)
Weighted SBP variability (SD)†	12.1 (3.4)	11.9 (3.1)	12.0 (3.0)	11.5 (2.4)	11.9 (3.2)	12.0 (3.2)	0.677	-0.2 (-1.2-0.8)	0.918	-0.1 (-1.4-1.3)	0.474	-0.6 (-2.3-1.1)	0.636	-0.2 (-1.3-0.8)	0.781	-0.1 (-1.1-0.9)
Weighted DBP variability (SD)†	10.2 (3.0)	10.0 (2.6)	9.8 (2.5)	10.2 (2.7)	10.1 (2.6)	10.0 (2.6)	0.723	-0.2 (-1.0-0.7)	0.499	-0.4 (-1.6-0.8)	0.997	0 (-1.5-1.5)	0.888	-0.1 (-0.9-0.8)	0.692	-0.2 (-1.0-0.7)
SBP variability (SD)	14.3 (3.2)	14.1 (3.3)	14.0 (3.2)	13.5 (3.0)	14.1 (3.3)	14.2 (3.3)	0.609	-0.3 (-1.2-0.7)	0.560	-0.4 (-1.7-0.9)	0.278	-0.9 (-2.5-0.7)	0.690	-0.2 (-1.2-0.8)	0.762	-0.2 (-1.2-0.8)
DBP variability (SD)	11.9 (3.0)	11.6 (2.9)	11.5 (3.0)	11.9 (2.9)	11.7 (2.9)	11.6 (3.0)	0.517	-0.29 (-1.17-0.59)	0.544	-0.39 (-1.65-0.87)	0.962	-0.04 (-1.59-1.52)	0.580	-0.26 (-1.19 (0.67)	0.475	-0.33 (-1.24-0.58)
SBP CV	12.0 (2.4)	11.5 (2.6)	11.2 (2.4)	10.9 (2.4)	11.6 (2.6)	11.6 (2.6)	0.241	-0.5 (-1.2-0.3)	0.119	-0.8 (-1.8-0.2)	0.091	-1.1 (-2.3-0.2)	0.404	-0.3 (-1.1-0.5)	0.368	-0.4 (-1.1-0.4)
DBP CV	16.7 (4.0)	16.7 (4.6)	16.7 (5.1)	17.3 (5.0)	16.6 (4.4)	16.6 (4.5)	0.942	-0.05 (-1.36-1.26)	0.980	-0.02 (-1.86-1.82)	0.613	0.56 (-1.64-2.77)	0.933	-0.06 (-1.39-1.27)	0.833	-0.14 (-1.46-1.18)
SBP load (%)‡	34.8 (20.2)	43.1 (26.2)	53.0 (26.3)	52.1 (27.1)	39.9 (25.5)	41.7 (25.9)	**0.014**	8.3 (1.7-14.9)	**<0.001**	18.2 (7.9-28.5)	**0.017**	17.3 (3.4-31.1)	0.148	5.1 (-1.8-12.1)	**0.046**	6.9 (0.1-13.7)
DBP load (%)‡	27.0 (18.1)	25.1 (16.6)	24.5 (17.5)	20.0 (25.0)*	25.3 (16.3)	23.0 (21.0)*	0.458	-1.9 (-7.0-3.2)	0.503	-2.5 (-10.0-5.0)	0.296	-4.0 (-12.0-4.0)*	0.525	-1.7 (-7.0-3.6)	0.555	-1.6 (-6.7-3.6)
Maximum SBP (mmHg)	154.2 (14.7)	154.5 (15.8)	158.1 (16.2)	155.0 (15.6)	153.4 (15.5)	154.4 (15.8)	0.884	0.3 (-4.3-4.9)	0.229	3.9 (-2.5-10.3)	0.833	0.8 (-6.9-8.6)	0.741	-0.8 (-5.6-4.0)	0.912	0.3 (-4.4-5.0)
Minimum SBP (mmHg)	89.7 (10.0)	92.1 (12.0)	96.0 (12.9)	94.6 (11.9)	90.8 (11.5)	91.7 (12.0)	0.175	2.4 (-1.1-5.7)	**0.008**	6.3 (1.7-10.9)	0.076	4.9 (-0.5-10.3)	0.534	1.1 (-2.3-4.5)	0.266	2.0 (-1.5-5.4)
Maximum DBP (mmHg)	100.8 (12.1)	97.8 (10.6)	97.9 (10.9)	98.6 (12.8)	97.8 (10.5)	97.7 (10.2)	0.081	-2.9 (-6.2-0.4)	0.251	-2.8 (-7.7-2.0)	0.500	-2.2 (-8.5-4.2)	0.095	-3.0 (-6.4-0.5)	0.073	-3.0 (-6.4-0.3)
Minimum DBP (mmHg)	46.7 (6.4)	46.2 (8.0)	46.2 (8.5)	43.7 (7.7)	46.3 (7.8)	46.6 (8.0)	0.665	-0.5 (-2.5-1.6)	0.738	-0.5 (-3.5-2.5)	0.091	-3.0 (-6.5-0.5)	0.693	-0.4 (-2.6-1.7)	0.951	-0.1 (-2.2-2.1)
SBP dip (%)	11.5 (6.9)	11.2 (6.0)	9.5 (6.1)	11.0 (5.1)	11.7 (5.9)	11.2 (6.1)	0.738	-0.3 (-2.2-1.6)	0.169	-1.9 (-4.7-0.8)	0.781	-0.5 (-3.9-2.9)	0.838	0.2 (-1.8-2.2)	0.767	-0.3 (-2.2-1.7)
DBP dip (%)	14.8 (9.7)	15.2 (8.3)	15.4 (9.3)	16.3 (9.9)	15.1 (8.0)	15.0 (8.1)	0.793	0.4 (-2.3-3.0)	0.776	0.6 (-3.4-4.6)	0.576	1.4 (-3.7-6.5)	0.841	0.3 (-2.4-3.0)	0.891	0.2 (-2.5-2.8)
**Daytime**	*N = 71*	*N = 154*	*N = 38*	*N = 21*	*N = 116*	*N = 133*										
SBP (mmHg)	123.5 (7.6)	126.1 (8.9)	128.2 (9.8)	128.1 (9.3)	125.4 (8.5)	125.8 (8.8)	**0.035**	2.6 (0.2-5.0)	**0.012**	4.7 (1.1-8.4)	**0.022**	4.6 (0.7-8.6)	0.126	1.9 (-0.5-4.3)	0.068	2.3 (1.2-4.7)
DBP (mmHg)	73.9 (6.4)	73.4 (6.1)	73.4 (6.9)	73.9 (7.1)	73.4 (5.8)	73.3 (5.9)	0.557	-0.52 (-2.26-1.22)	0.693	-0.52 (-3.13-2.09)	0.995	-0.01 (-3.24-3.22)	0.568	-0.52 (-2.31-1.27)	0.502	-0.60 (-2.36-1.16)
PP (mmHg)	49.6 (5.0)	52.7 (7.4)	54.8 (8.7)	54.2 (9.6)	52.1 (6.8)	52.5 (7.0)	**<0.001**	3.1 (1.5-4.8)	**0.001**	5.2 (2.1-8.2)	**0.047**	4.6 (0.1-9.1)	**0.006**	2.4 (0.7-4.1)	**<0.001**	2.9 (1.2-4.6)
HR (bpm)	85.4 (7.3)	84.9 (8.3)	85.8 (8.2)	88.6 (6.8)	84.5 (8.3)	84.3 (8.3)	0.639	-0.5 (-2.8-1.7)	0.770	0.5 (-2.6-3.5)	0.073	3.2 (-0.3-6.8)	0.472	-0.9 (-3.2-1.5)	0.336	-1.1 (-3.5-1.2)
SBP variability (SD)	12.7 (3.5)	12.7 (3.7)	12.8 (3.7)	12.0 (3.3)	12.7 (3.8)	12.8 (3.8)	0.959	0.03 (-1.01-1.07)	0.851	0.14 (-1.30-1.57)	0.453	-0.65 (-2.37-1.07)	0.987	-0.01 (-1.10-1.08)	0.806	0.13 (-0.94-1.21)
DBP variability (SD)	10.7 (3.4)	10.5 (3.3)	10.4 (3.5)	10.4 (3.9)	10.5 (3.2)	10.5 (3.2)	0.686	-0.2 (-1.1-0.8)	0.709	-0.3 (-1.6-1.1)	0.741	-0.3 (-2.0-1.4)	0.730	-0.2 (-1.2-0.8)	0.712	-0.2 (-1.1-0.8)
SBP CV	10.2 (2.6)	10.1 (2.8)	10.0 (2.7)	9.4 (2.4)	10.1 (2.9)	10.2 (2.9)	0.683	-0.2 (-0.9-0.6)	0.653	-0.2 (-1.3-0.8)	0.195	-0.8 (-2.1-0.4)	0.745	-0.1 (-1.0-0.7)	0.891	-0.1 (-0.9-0.8)
DBP CV	14.5 (4.4)	14.4 (4.6)	14.4 (5.0)	14.1 (5.2)	14.4 (4.5)	14.4 (4.5)	0.920	-0.07 (-1.34-1.21)	0.911	-0.10 (-1.93-1.72)	0.780	-0.32 (-2.56-1.93)	0.937	-0.05 (-1.37-1.27)	0.968	-0.03 (-1.32-1.27)
SBP load (%)‡	35.1 (21.0)	43.4 (27.2)	52.2 (27.7)	55.4 (26.4)	40.5 (26.6)	41.5 (27.0)	**0.014**	8.3 (1.7-14.8)	**0.002**	17.0 (6.8-27.3)	**<0.001**	20.3 (9.3-31.3)	0.125	5.4 (-1.5-12.3)	0.063	6.4 (-0.4-13.1)
DBP load (%)‡	19.0 (28.0)*	17.5 (23.0)*	19.5 (25.0)*	20.0 (24.0)*	17.0 (23.0)*	17.0 (23.0)*	0.671	-1.0 (-6.0-4.0)*	0.874	0 (-8.0-6.0)*	0.834	0 (-10.0-8.0)*	0.641	-1.0 (-6.0-4.0)*	0.672	-1.0 (-6.0-4.0)*
Maximum SBP (mmHg)	152.9 (15.9)	153.8 (16.0)	155.8 (16.9)	154.2 (15.8)	153.1 (15.7)	153.7 (16.1)	0.697	0.9 (-3.6-5.4)	0.372	2.9 (-3.6-9.4)	0.728	1.4 (-6.5-9.2)	0.924	0.2 (-4.5-4.9)	0.729	0.8 (-3.8-5.5)
Minimum SBP (mmHg)	99.6 (9.5)	99.7 (13.3)	102.5 (14.1)	103.0 (13.8)	98.8 (13.0)	99.2 (13.2)	0.939	0.1 (-3.0-3.2)	0.262	2.9 (-2.2-8.0)	0.294	3.4 (-3.2-10.0)	0.634	-0.8 (-4.0-2.5)	0.801	-0.4 (-3.6-2.8)
Maximum DBP (mmHg)	99.2 (12.8)	97.1 (11.2)	96.8 (11.5)	97.0 (13.8)	97.1 (11.1)	97.1 (10.7)	0.211	-2.1 (-5.4-1.2)	0.353	-2.3 (-7.2-2.6)	0.497	-2.2 (-8.6-4.2)	0.252	-2.0 (-5.5-1.5)	0.218	-2.1 (-5.4-1.2)
Minimum DBP (mmHg)	53.1 (8.8)	52.6 (9.3)	52.6 (9.5)	51.8 (10.0)	52.5 (9.3)	52.7 (9.2)	0.689	-0.5 (-3.1-2.1)	0.786	-0.5 (-4.1-3.1)	0.562	-1.3 (-5.8-3.2)	0.696	-0.5 (-3.2-2.2)	0.764	-0.4 (-3.0-2.2)
**Nighttime**	*N = 69*	*N = 154*	*N = 38*	*N = 19*	*N = 116*	*N = 135*										
SBP (mmHg)	109.0 (9.1)	112.1 (10.7)	115.7 (11.6)	113.6 (10.8)	111.0 (10.2)	111.9 (10.7)	**0.033**	3.2 (0.3-6.1)	**0.001**	6.8 (2.7-10.8)	0.064	4.6 (-0.3-9.5)	0.181	2.0 (-0.9-4.9)	0.051	3.0 (0-6.0)
DBP (mmHg)	62.8 (7.6)	62.2 (7.3)	61.5 (8.1)	61.0 (8.2)	62.4 (7.0)	62.3 (7.1)	0.548	-0.6 (1.1- -2.7)	0.410	-1.3 (-4.4-1.8)	0.371	-1.8 (-5.8-2.2)	0.698	-0.4 (-2.6-1.7)	0.658	-0.5 (-2.6-1.7)
PP (mmHg)	46.0 (7.8)	49.7 (8.7)	53.2 (6.7)	52.6 (10.2)	48.6 (8.0)	49.3 (8.4)	**0.003**	3.7 (1.3-6.1)	**<0.001**	7.1 (3.7-10.6)	**0.003**	6.6 (2.3-10.9)	**0.036**	2.6 (0.2-4.9)	**0.008**	3.3 (0.9-5.6)
HR (bpm)	70.5 (9.1)	70.0 (9.2)	70.6 (9.5)	72.4 (9.7)	69.8 (9.2)	69.7 (9.1)	0.751	-0.4 (-3.1-2.2)	0.945	0.1 (-3.6-3.8)	0.412	2.0 (-2.8-6.7)	0.664	-0.6 (-3.4-2.1)	0.574	-0.8 (-3.4-1.9)
SBP variability (SD)	11.4 (4.9)	10.6 (4.1)	10.8 (4.3)	10.9 (3.7)	10.5 (4.0)	10.5 (4.2)	0.178	-0.9 (-2.1-0.4)	0.509	-0.6 (-2.5-1.3)	0.681	-0.5 (-2.9-1.9)	0.166	-0.9 (-2.2-0.4)	0.170	-0.9 (-2.2-0.4)
DBP variability (SD)	8.5 (4.0)*	8.6 (4.5)*	8.5 (5.8)*	8.9 (5.5)*	8.8 (3.8)*	8.5 (4.1)*	0.835	0.10 (-0.79-0.96)*	0.946	-0.09 (-1.49-1.29)*	0.296	1.03 (-1.02-2.88)*	0.770	0.15 (-0.75-1.01)*	0.979	-0.01 (-0.89-0.85)*
SBP CV	10.4 (4.0)	9.4 (3.5)	9.3 (3.5)	9.6 (3.1)	9.5 (3.5)	9.4 (3.6)	0.072	-1.0 (-2.0-0.1)	0.174	-1.1 (-2.6-0.5)	0.427	-0.8 (-2.8-1.2)	0.101	-0.9 (-2.0-0.2)	0.076	-1.0 (-2.1-0.1)
DBP CV	14.0 (6.3)*	14.2 (6.5)*	14.1 (7.8)*	15.2 (14.4)*	14.4 (6.0)*	14.1 (6.5)*	0.959	0.04 (-1.36-1.48)*	0.933	-0.06 (-2.40-2.14)*	0.250	1.86 (-1.16-4.94)*	0.919	0.08 (-1.37-1.50)*	0.821	-0.16 (-1.59-1.27)*
SBP load (%)‡	32.9 (27.5)	41.1 (31.0)	53.3 (31.7)	47.2 (31.0)	37.2 (29.8)	40.3 (31.0)	0.059	8.2 (-0.3-16.8)	**<0.001**	20.4 (8.7-32.0)	0.055	14.3 (-0.3-28.8)	0.336	4.2 (4.4-12.9)	0.096	7.4 (-1.3-16.1)
DBP load (%)‡	34.0 (26.0)	35.7 (26.2)	32.3 (26.1)	29.2 (23.4)	36.8 (26.3)	36.6 (26.5)	0.655	1.7 (-5.8-9.2)	0.743	-1.7 (-12.2-8.7)	0.469	-4.8 (-17.9-8.3)	0.480	2.8 (-5.0-10.7)	0.504	2.6 (-5.1-10.3)
Maximum SBP (mmHg)	126.7 (14.8)	129.0 (14.5)	132.7 (15.5)	131.5 (15.4)	127.8 (14.0)	128.7 (14.3)	0.271	2.3 (-1.8-6.5)	0.050	6.0 (0-12.1)	0.221	4.8 (-2.9-12.5)	0.607	1.1 (-3.2-5.4)	0.356	2.0 (-2.2-6.2)
Minimum SBP (mmHg)	90.9 (10.0)	96.5 (11.2)	100.4 (11.8)	97.6 (10.3)	95.2 (10.8)	96.3 (11.3)	**<0.001**	5.6 (2.5-8.7)	**<0.001**	9.5 (5.2-13.8)	**0.012**	6.7 (1.5-11.9)	**0.008**	4.3 (1.1-7.4)	**0.001**	5.4 (2.2-8.6)
Maximum DBP (mmHg)	75.0 (12.0)*	76.0 (15.0)*	77.0 (13.0)	79.7 (12.3)	77.9 (12.4)	77.4 (12.1)	0.947	0 (-3.0-3.0)*	0.607	-1.0 (-6.0-4.0)*	0.911	0 (-6.0-8.0)*	0.888	0 (-3.0-3.0)*	0.919	0 (-3.0-3.0)*

Minimum DBP (mmHg)	49.4 (7.6)	48.7 (8.6)	48.3 (9.5)	46.5 (9.3)	48.8 (8.4)	49.0 (8.5)	0.550	-0.7 (-3.1-1.7)	0.529	-1.1 (-4.4-2.3)	0.163	-2.9 (-7.0-1.2)	0.620	-0.6 (-3.0-1.8)	0.734	-0.4 (-2.8-2.0)
**Height specific z-scores**																
24-hour blood pressure	*N = 63*	*N = 143*	*N = 35*	*N = 19*	*N = 108*	*N = 124*										
SBP	1.26 (0.99)	1.60 (1.24)	2.07 (1.29)	1.99 (1.26)	1.45 (1.19)	1.54 (1.23)	0.058	0.34 (-0.01-0.69)	**<0.001**	0.81 (0.34-1.27)	**0.010**	0.73 (0.18-1.28)	0.295	0.19 (-0.16-0.54)	0.122	0.28 (-0.08-0.63)
DBP	0.80 (0.99)	0.62 (1.05)	0.60 (1.09)	0.57 (1.11)	0.63 (1.04)	0.63 (1.04)	0.256	-0.18 (-0.49-0.13)	0.358	-0.20 (-0.63-0.23)	0.392	-0.23 (-0.76-0.30)	0.294	-0.17 (-0.49-0.15)	0.286	-0.17 (-0.48-0.14)
HR	-0.27 (0.79)	-0.32 (0.91)	-0.31 (0.97)	-0.10 (0.90)	-0.32 (0.89)	-0.35 (0.90)	0.699	-0.05 (-0.31-0.21)	0.821	-0.04 (-0.40-0.32)	0.423	0.17 (-0.25-0.60)	0.689	-0.05 (-0.32-0.21)	0.527	-0.09 (-0.35-0.18)
Daytime	*N = 71*	*N = 153*	*N = 38*	*N = 21*	*N = 115*	*N = 132*										
SBP	1.14 (1.06)	1.41 (1.22)	1.80 (1.30)	1.88 (1.17)	1.28 (1.17)	1.34 (1.22)	0.109	0.27 (-0.06-0.60)	**0.005**	0.66 (0.20-1.11)	**0.007**	0.74 (0.20-1.28)	0.399	0.14 (-0.19-0.48)	0.253	0.20 (-0.14-0.53)
DBP	0.34 (1.06)	0.22 (1.04)	0.24 (1.18)	0.34 (1.24)	0.22 (0.99)	0.20 (1.00)	0.452	-0.11 (-0.41-0.18)	0.665	-0.10 (-0.53-0.34)	0.984	0.01 (-0.54-0.55)	0.440	-0.12 (-0.42-0.18)	0.383	-0.13 (-0.43-0.17)
HR	-0.46 (0.73)	-0.48 (0.87)	-0.48 (0.90)	-0.25 (0.80)	-0.47 (0.87)	-0.51 (0.88)	0.870	-0.02 (-0.25-0.22)	0.883	-0.02 (-0.34-0.29)	0.262	0.21 (-0.16-0.58)	0.882	-0.02 (-0.26-0.22)	0.648	-0.06 (-0.30-0.19)
Nighttime	*N = 69*	*N = 153*	*N = 38*	*N = 19*	*N = 115*	*N = 134*										
SBP	1.08 (1.08)	1.39 (1.18)	1.90 (1.26)	1.71 (1.17)	1.22 (1.10)	1.34 (1.17)	0.069	0.30 (-0.02-0.63)	**<0.001**	0.81 (0.35-1.27)	**0.031**	0.62 (0.06-1.19)	0.418	0.14 (-0.19-0.46)	0.129	0.26 (-0.08-0.59)
DBP	1.14 (1.20)	1.07 (1.14)	0.98 (1.25)	0.89 (1.28)	1.10 (1.10)	1.09 (1.12)	0.662	-0.07 (-0.40-0.26)	0.507	-0.16 (-0.65-0.32)	0.433	-0.25 (-0.87-0.38)	0.801	-0.04 (-0.39-0.30)	0.775	-0.05 (-0.38-0.29)
HR	-0.08 (0.97)	-0.10 (1.01)	-0.11 (1.09)	0.05 (1.08)	-0.10 (0.99)	-0.12 (1.00)	0.877	-0.02 (-0.31-0.26)	0.863	-0.04 (-0.44-0.37)	0.619	0.13 (-0.38-0.64)	0.904	-0.02 (-0.31-0.28)	0.766	-0.04 (-0.33-0.25)
**Age specific z-scores**																
24-hour blood pressure	*N = 63*	*N = 144*	*N = 35*	*N = 19*	*N = 109*	*N = 125*										
SBP	1.13 (0.89)	1.41 (1.08)	1.74 (1.21)	1.57 (1.22)	1.30 (1.02)	1.38 (1.07)	0.078	0.28 (-0.03-0.58)	**0.005**	0.61 (0.19-1.04)	0.158	0.44 (-0.18-1.06)	0.280	0.17 (-0.14-0.47)	0.110	0.25 (-0.06-0.56)
DBP	0.07 (0.01)	0.07 (0.01)	0.07 (0.01)*	0.07 (0.01)	0.07 (0.01)*	0.07 (0.01)	0.147	0 (0-0)	0.311	0 (0-0)*	0.145	0 (0-0)	0.140	0 (0-0)	0.214	0 (0-0)
HR	-0.22 (0.81)	-0.27 (0.94)	-0.18 (0.99)	0.14 (0.87)	-0.30 (0.93)	-0.34 (0.94)	0.679	-0.06 (-0.33-0.21)	0.837	0.04 (-0.33-0.41)	0.106	0.35 (-0.08-0.79)	0.536	-0.09 (-0.36-0.19)	0.392	-0.12 (-0.39-0.15)
Daytime	*N = 71*	*N = 154*	*N = 38*	*N = 21*	*N = 116*	*N = 133*										
SBP	1.02 (0.96)	1.26 (1.09)	1.54 (1.22)	1.50 (1.15)	1.17 (1.03)	1.22 (1.08)	0.118	0.24 (-0.06-0.53)	**0.017**	0.52 (0.09-0.94)	0.059	0.48 (-0.02-0.97)	0.342	0.14 (-0.15-0.44)	0.197	0.20 (-0.10-0.50)
DBP	0.36 (1.08)	0.25 (1.01)	0.25 (1.16)	0.33 (1.22)	0.25 (0.96)	0.24 (0.98)	0.476	-0.11 (-0.40-0.19)	0.635	-0.11 (-0.55-0.34)	0.938	-0.02 (-0.57-0.53)	0.487	-0.11 (-0.40-0.19)	0.425	-0.12 (-0.41-0.17)
HR	-0.43 (0.77)	-0.43 (0.91)	-0.34 (0.93)	0.01 (0.76)	-0.46 (0.91)	-0.50 (0.92)	0.973	0 (-0.25-0.24)	0.605	0.09 (-0.24-0.42)	**0.023**	0.44 (0.06-0.82)	0.793	-0.03 (-0.29-0.22)	0.560	-0.07 (-0.33-0.18)
Nighttime	*N = 69*	*N = 154*	*N = 38*	*N = 19*	*N = 116*	*N = 135*										
SBP	1.03 (1.03)	1.33 (1.12)	1.75 (1.23)	1.50 (1.17)	1.19 (1.06)	1.31 (1.12)	0.061	0.30 (-0.01-0.61)	**0.002**	0.72 (0.28-1.16)	0.093	0.47 (-0.08-1.01)	0.312	0.16 (-0.15-0.47)	0.089	0.28 (-0.04-0.59)
DBP	1.07 (1.07)	1.02 (1.07)	0.92 (1.15)	0.85 (1.19)	1.05 (1.04)	1.04 (1.05)	0.742	-0.05 (-0.36-0.25)	0.499	-0.15 (-0.59-0.29)	0.451	-0.22 (-0.78-0.35)	0.910	-0.02 (-0.33-0.30)	0.859	-0.03 (-0.34-0.28)
HR	-0.03 (0.97)	-0.06 (1.01)	-0.02 (1.09)	0.23 (1.05)	-0.07 (0.99)	-0.10 (1.00)	0.877	-0.02 (-0.31-0.26)	0.935	0.02 (-0.39-0.42)	0.313	0.26 (-0.25-0.77)	0.813	-0.04 (-0.33-0.26)	0.672	-0.06 (-0.35-0.23)
**SBP dipping§**	*N = 63*	*N = 144*	*N = 35*	*N = 19*	*N = 109*	*N = 125*	**0.046**	-	**0.035**	-	0.063	-	0.112	-	0.128	-
Nondipping, n (%)	13 (20.6)	48 (33.3)	15 (42.9)	9 (47.4)	33 (30.3)	39 (31.2)										
Normal dipping, n (%)	37 (58.7)	84 (58.3)	18 (51.4)	10 (52.6)	66 (60.6)	74 (59.2)										
Extreme dipping, n (%)	7 (11.1)	7 (4.9)	0 (0)	0 (0)	7 (6.4)	7 (5.6)										
Reversed dipping, n (%)	6 (9.5)	5 (3.5)	2 (5.7)	0 (0)	3 (2.8)	5 (4.0)										
**DBP dipping§**	*N = 63*	*N = 144*	*N = 35*	*N = 19*	*N = 109*	*N = 125*	0.401	-	0.572	-	0.933	-	0.416	-	0.298	-
Nondipping, n (%)	10 (15.9)	27 (18.8)	9 (25.7)	4 (21.1)	18 (16.5)	23 (18.4)										
Normal dipping, n (%)	27 (42.9)	74 (51.4)	15 (42.9)	7 (36.8)	59 (54.1)	67 (53.6)										
Extreme dipping, n (%)	21 (33.3)	37 (25.7)	10 (28.6)	7 (36.8)	27 (24.8)	30 (24.0)										
Reversed dipping, n (%)	5 (7.9)	6 (4.2)	1 (2.9)	1 (5.3)	5 (4.6)	5 (4.0)										

Data is presented as mean (SD) unless stated otherwise. Independent Samples t Test for normally distributed numerical data, Mann-Whitney U Test for non-normal distribution and Pearson Chi-Square or Fischer's Exact Test for categorical data.

PE indicates pre-eclampsia; SBP, systolic blood pressure; DBP, diastolic blood pressure; SD, standard deviation; CV, coefficient of variation; HR, heart rate; PP, pulse pressure; bpm, beats per minute; IQR, interquartile range; CI, confidence interval; PWV, pulse wave velocity; dg, diagnosis.

*Median (IQR), median difference (95% CI)

†Weighted for the duration of daytime and nighttime

‡Proportion of measurements exceeding the height- and gender specific 95th percentile

§Nondipping: blood pressure decrease of 0% or greater, but less than 10%; normal dipping: blood pressure decrease of 10% or greater, but less than 20%; extreme dipping: blood pressure decrease of 20% or greater; reversed dipping: blood pressure increase during nighttime

Supplementary Table 5. Blood parameters	non-PE	PE	Early-onset *Diagnosis* *<34^0/7^ weeks*	Late-onset *Diagnosis* *≥34^0/7^ weeks*	Mean difference (95% CI) PE vs non-PE	Mean difference (95% CI) Early (dg) versus non-PE	Mean difference (95% CI) Late (dg) versus non-PE
**Laboratory**	*N = 55*	*N = 122*	*N = 28*	*N = 94*			
hs-CRP (mg/l)	0.21 (0.22)*	0.21 (0.34)*	0.29 (0.49)*	0.19 (0.27)*	0 (-0.01-0.03)*	0.06 (0-0.15)*	0 (-0.01-0.02)*
Creatinine (µmol/l)	43.5 (5.9)	45.0 (8.2)	45.1 (8.3)	45.0 (8.2)	1.5 (-0.6-3.7)	1.7 (-1.5-4.8)	1.5 (-0.8-3.8)
Glucose (mmol/l)†	5.4 (0.4)*	5.4 (0.4)*	5.4 (0.4)	5.3 (0.4)	0 (-0.1-0.1)*	0.1 (-0.1-0.3)*	0 (-0.1-0.1)*
Insulin (mU/l)	9.6 (7.5)*	10.1 (7.8)*	11.1 (11.0)*	9.9 (6.7)*	0.3 (-1.4-1.9)*	1.3 (-1.2-4.5)*	0 (-1.7-1.6)*
HOMA-IR	2.3 (1.7)*	2.4 (2.0)*	3.0 (2.6)*	2.3 (1.7)*	0.10 (-0.31-0.51)*	0.39 (-0.29-1.22)*	0.03 (-0.39-0.42)*
Alanine aminotransferase (U/l)	18 (9)*	17 (7)*	17 (9)*	16 (7)*	-1.0 (-3.0-1.0)*	-1.0 (-3.0-2.0)*	-1.0 (-3.0-1.0)*
Uric acid (µmol/l)	238.4 (64.8)	250.3 (56.2)	259.8 (52.3)	247.5 (57.3)	12.0 (-7.0-30.9)	21.4 (-6.7-49.6)	9.1 (-11.1-29.4)
Total cholesterol (mmol/l)†	4.23 (0.72)	4.23 (0.75)	4.02 (0.56)	4.30 (0.78)	0.01 (-0.23-0.24)	-0.21 (-0.52-0.10)	0.07 (-0.19-0.33)
HDL (mmol/l)†	1.47 (0.39)*	1.48 (0.47)*	1.42 (0.22)	1.54 (0.35)	0 (-0.10-0.09)*	-0.08 (-0.19-0.04)*	0.03 (-0.08-0.13)*
LDL (mmol/l)†	2.52 (0.75)	2.63 (0.72)	2.61 (0.67)	2.64 (0.73)	0.11 (-0.12-0.34)	0.09 (-0.25-0.42)	0.12 (-0.13-0.37)
Triglycerides (mmol/l)†	0.67 (0.45)*	0.71 (0.36)*	0.69 (0.27)*	0.72 (0.39)*	0.02 (-0.06-0.10)*	-0.01 (-0.14-0.10)*	0.03 (-0.06-0.12)*

Data is presented as mean (SD) unless stated otherwise. Independent Samples t Test for normally distributed numerical data and Mann-Whitney U Test for non-normal distribution. PE indicates pre-eclampsia; SD, standard deviation; IQR, interquartile range; CI, confidence interval; hs-CRP, high-sensitivity C-reactive protein; HDL, high-density lipoprotein; LDL, low-density lipoprotein; HOMA-IR calculated as (glucose (mmol/l) x insulin (mU/l))/22; dg, diagnosis.

*Median (IQR), median difference (95% CI)

†Fasting levels

Supplementary Table 6. Results of univariate linear regressions for pre-eclamptic children's ambulatory blood pressure and pulse pressure and pulse wave velocities

		**24-hour systolic blood pressure**				**24-hour pulse pressure**				**Daytime systolic blood pressure**				**Daytime pulse pressure**			
		B (95% CI)	Standardized B	R^2	*p*	B (95% CI)	Standardized B	R^2	*p*	B (95% CI)	Standardized B	R^2	*p*	B (95% CI)	Standardized B	R^2	*p*
Maternal age at delivery (years)	Pregnancy	0.15 (-0.16-0.46)	0.079	0.006	0.344	0.09 (-0.18-0.35)	0.055	0.003	0.516	0.12 (-0.18-0.42)	0.065	0.004	0.425	0.09 (-0.16-0.34)	0.055	0.003	0.494
Maternal pre-pregnancy BMI (kg/m^2)		0.16 (-0.16-0.48)	0.084	0.007	0.318	0.45 (0.18-0.72)	0.270	0.073	**0.001**	0.13 (-0.18-0.45)	0.068	0.005	0.403	0.35 (0.10-0.61)	0.217	0.047	**0.007**
Primiparous (0 = yes, 1 = no)		4.37 (1.01-7.73)	0.211	0.044	**0.011**	5.10 (2.27-7.92)	0.287	0.082	**<0.001**	3.28 (-0.07-6.62)	0.155	0.024	0.055	4.33 (1.60-7.06)	0.247	0.061	**0.002**
Gestational weeks at delivery		-0.418 (-0.834- -0.002)	-0.165	0.027	**0.049**	-0.34 (-0.70-0.02)	-0.156	0.024	0.063	-0.39 (-0.80-0.02)	-0.151	0.023	0.061	-0.25 (-0.60-0.09)	-0.118	0.014	0.145
Premature (0 = no, 1 = yes)		4.58 (1.58-7.58)	0.246	0.060	**0.003**	4.67 (2.13-7.21)	0.291	0.085	**<0.001**	4.11 (1.16-7.05)	0.218	0.048	**0.007**	3.70 (1.27-6.13)	0.237	0.056	**0.003**
Birth height (cm)		-0.18 (-0.49-0.13)	-0.096	0.009	0.253	-0.19 (-0.45-0.08)	-0.115	0.013	0.170	-0.20 (-0.51-0.10)	-0.106	0.011	0.194	-0.15 (-0.40-0.11)	-0.094	0.009	0.250
Birth height z-score		-0.07 (-1.17-1.03)	-0.010	0.000	0.906	-0.44 (-1.37-0.50)	-0.077	0.006	0.361	-0.22 (-1.29-0.85)	-0.033	0.001	0.686	-0.46 (-1.35-0.42)	-0.084	0.007	0.301
Birth weight (g)		-0.002 (-0.003-0)	-0.179	0.032	**0.032**	-0.001 (-0.003-0)	-0.141	0.020	0.091	-0.002 (-0.003-0)	-0.164	0.027	**0.042**	-0.001 (-0.002-0)	-0.109	0.012	0.179
Birth weight z-score		-0.87 (-1.97-0.24)	-0.129	0.017	0.125	-0.52 (-1.48-0.43)	-0.091	0.008	0.280	-0.76 (-1.84-0.33)	-0.111	0.012	0.170	-0.43 (-1.34-0.47)	-0.076	0.006	0.347
SGA (0 = no, 1 = yes)		4.54 (0.63-8.46)	0.189	0.036	**0.023**	2.60 (-0.79-5.99)	0.126	0.016	0.132	4.24 (0.57-7.91)	0.182	0.033	**0.024**	1.84 (-1.24-4.93)	0.095	0.009	0.240
Birth head circumference (cm)		-0.33 (-0.77-0.12)	-0.123	0.015	0.146	-0.26 (-0.64-0.13)	-0.111	0.012	0.190	-0.32 (-0.75-0.11)	-0.121	0.015	0.142	-0.21 (-0.57-0.16)	-0.091	0.008	0.267
Birth head circumference z-score		-0.75 (-1.90-0.41)	-0.107	0.012	0.203	-0.65 (-1.64-0.35)	-0.108	0.012	0.200	-0.65 (-1.75-0.45)	-0.095	0.009	0.245	-0.60 (-1.53-0.33)	-0.105	0.011	0.203
Sex (0 = female, 1 = male)	Sex	0.83 (-2.11-3.77)	0.047	0.002	0.578	0.23 (-2.29-2.76)	0.015	0.000	0.857	0.30 (-2.56-3.15)	0.017	0.000	0.838	0.34 (-2.03-2.71)	0.023	0.001	0.778
Age (years)	Age	-0.08 (-1.37-1.22)	-0.010	0.000	0.909	0.56 (0.55-1.66)	0.083	0.007	0.323	-0.19 (-1.45-1.07)	-0.024	0.001	0.771	0.51 (-0.54-1.55)	0.078	0.006	0.337
Body height (cm)	Anthropometrics	0.06 (-0.08-0.20)	0.069	0.005	0.415	0.15 (0.03-0.26)	0.204	0.041	**0.014**	0.04 (-0.09-0.18)	0.052	0.003	0.522	0.108 (-0.003-0.220)	0.153	0.024	0.057
Body weight (kg)		0.08 (-0.04-0.20)	0.114	0.013	0.172	0.19 (0.09-0.28)	0.308	0.095	**<0.001**	0.04 (-0.08-0.15)	0.053	0.003	0.517	0.14 (0.04-0.23)	0.230	0.053	**0.004**
Body surface area (m^2)		4.27 (-1.92-10.45)	0.114	0.013	0.175	9.86 (4.78-14.94)	0.306	0.094	**<0.001**	2.26 (-3.89-8.41)	0.059	0.003	0.469	7.37 (2.39-12.35)	0.231	0.053	**0.004**
Lean body mass (kg)		0.15 (-0.04-0.34)	0.129	0.017	0.126	0.29 (0.13-0.45)	0.294	0.087	**<0.001**	0.11 (-0.09-0.29)	0.088	0.008	0.277	0.23 (0.08-0.39)	0.239	0.057	**0.003**
Skeletal muscle mass (kg)		0.25 (-0.07-0.58)	0.131	0.017	0.120	0.49 (0.23-0.76)	0.294	0.087	**<0.001**	0.18 (-0.14-0.50)	0.090	0.008	0.266	0.40 (0.14-0.66)	0.240	0.058	**0.003**
Height z-score		0.84 (-0.40-2.08)	0.111	0.012	0.184	1.33 (0.28-2.38)	0.205	0.042	**0.014**	0.75 (-0.47-1.97)	0.099	0.010	0.224	0.89 (-0.11-1.90)	0.141	0.020	0.082
Weight z-score (height)		1.08 (-0.22-2.39)	0.136	0.019	0.104	2.21 (1.14-3.28)	0.324	0.105	**<0.001**	0.47 (-0.81-1.75)	0.059	0.003	0.467	1.67 (0.64-2.70)	0.251	0.063	**0.002**
Weight z-score (age)		1.38 (0.09-2.67)	0.174	0.030	**0.036**	2.44 (1.39-3.49)	0.360	0.130	**<0.001**	0.89 (-0.38-2.17)	0.111	0.012	0.169	1.76 (0.73-2.79)	0.264	0.070	**<0.001**
Waist circumference (cm)	Adiposity	0.14 (0.01-0.27)	0.175	0.031	**0.036**	0.21 (0.10-0.32)	0.310	0.096	**<0.001**	0.07 (-0.06-0.20)	0.087	0.008	0.282	0.15 (0.05-0.26)	0.224	0.050	**0.005**
Waist-hip ratio (no unit)		30.87 (8.55-53.20)	0.224	0.050	**0.007**	18.47 (-0.94-37.88)	0.156	0.024	0.062	21.34 (-1.11-43.78)	0.151	0.023	0.062	13.66 (-5.07-32.38)	0.116	0.013	0.152
BMI (kg/m^2)		0.34 (-0.06-0.74)	0.139	0.019	0.097	0.68 (0.35-1.00)	0.324	0.105	**<0.001**	0.15 (-0.25-0.54)	0.059	0.003	0.467	0.50 (0.18-0.82)	0.241	0.058	**0.003**
BMI z-score		1.301 (0.002-2.600)	0.164	0.027	0.050	2.50 (1.45-3.55)	0.367	0.135	**<0.001**	0.72 (-0.57-2.00)	0.089	0.008	0.271	1.86 (0.84-2.89)	0.280	0.078	**<0.001**
Fat mass (kg)		0.11 (-0.11-0.33)	0.084	0.007	0.321	0.30 (0.12-0.48)	0.266	0.071	**0.001**	0.01 (-0.21-0.23)	0.008	0.000	0.921	0.19 (0.02-0.37)	0.172	0.030	**0.033**
Fat percentage (%)		0.06 (-0.11-0.22)	0.057	0.003	0.500	0.21 (0.08-0.35)	0.253	0.064	**0.002**	-0.01 (-0.17-0.15)	-0.012	0.000	0.882	0.133 (0.004-0.263)	0.163	0.027	**0.044**
SBP (mmHg)	Office BP	0.42 (0.29-0.55)	0.470	0.221	**<0.001**	0.32 (0.20-0.43)	0.414	0.171	**<0.001**	0.43 (0.30-0.55)	0.478	0.228	**<0.001**	0.29 (0.18-0.40)	0.392	0.154	**<0.001**
DBP (mmHg)		0.25 (0.01-0.50)	0.167	0.028	**0.046**	-0.04 (-0.26-0.17)	-0.033	0.001	0.698	0.34 (0.11-0.57)	0.233	0.054	**0.004**	-0.03 (-0.23-0.16)	-0.027	0.001	0.739
PP (mmHg)		0.38 (0.20-0.55)	0.339	0.115	**<0.001**	0.43 (0.29-0.58)	0.450	0.202	**<0.001**	0.39 (0.22-0.56)	0.346	0.119	**<0.001**	0.42 (0.29-0.56)	0.448	0.201	**<0.001**
HR (bpm)		-0.01 (-0.15-0.14)	-0.008	0.000	0.929	-0.05 (-0.17-0.07)	-0.068	0.005	0.425	-0.05 (-0.19-0.10)	-0.055	0.003	0.508	-0.07 (-0.19-0.05)	-0.096	0.009	0.245
SBP z-score		4.82 (3.20-6.43)	0.447	0.200	**<0.001**	3.08 (1.61-4.55)	0.331	0.110	**<0.001**	5.03 (3.50-6.56)	0.470	0.220	**<0.001**	2.91 (1.54-4.27)	0.325	0.106	**<0.001**
DBP z-score		2.523 (0.004-5.042)	0.166	0.027	0.050	-0.88 (-3.08-1.33)	-0.067	0.004	0.433	3.27 (0.92-5.62)	0.220	0.048	**0.007**	-0.81 (-2.82-1.19)	-0.065	0.004	0.424
Carotid-femoral PWV (m/s)	PWV	1.20 (-0.70-3.09)	0.107	0.011	0.213	0.06 (-1.60-1.73)	0.007	0.000	0.940	0.79 (-1.08-2.66)	0.069	0.005	0.403	0.11 (-1.47-1.70)	0.012	0.000	0.888
Carotid-radial PWV (m/s)		1.06 (-0.16-2.27)	0.147	0.022	0.087	-0.17 (-1.24-0.91)	-0.026	0.000	0.762	0.90 (-0.29-2.10)	0.123	0.015	0.137	-0.28 (-1.29-0.73)	-0.045	0.002	0.587
Central SBP (mmHg)		0.30 (0.19-0.41)	0.406	0.165	**<0.001**	0.31 (0.22-0.41)	0.485	0.235	**<0.001**	0.29 (0.18-0.40)	0.387	0.150	**<0.001**	0.28 (0.19-0.37)	0.456	0.208	**<0.001**
Central DBP (mmHg)		0.29 (0.04-0.54)	0.189	0.036	**0.026**	-0.03 (-0.25-0.20)	-0.021	0.000	0.806	0.35 (0.12-0.58)	0.239	0.057	**0.003**	-0.02 (-0.22-0.18)	-0.019	0.000	0.816
Central PP (mmHg)		0.25 (0.13-0.38)	0.329	0.108	**<0.001**	0.35 (0.25-0.45)	0.518	0.268	**<0.001**	0.21 (0.09-0.33)	0.274	0.075	**<0.001**	0.31 (0.22-0.40)	0.479	0.230	**<0.001**
SBP at first antenatal visit (mmHg)	Maternal BP	0.133 (0.002-0.263)	0.168	0.028	**0.047**	0.06 (-0.06-0.17)	0.083	0.007	0.326	0.11 (-0.01-0.24)	0.146	0.021	0.074	0.03 (-0.07-0.14)	0.049	0.002	0.553
Highest SBP before 20 gestwk (mmHg)		0.08 (-0.05-0.21)	0.126	0.016	0.217	0.06 (-0.06-0.18)	0.105	0.011	0.303	0.05 (-0.08-0.17)	0.073	0.005	0.466	0.03 (-0.08-0.14)	0.054	0.003	0.591
Highest SBP from 20 gestwk to childbirth (mmHg)		0.14 (0.04-0.24)	0.264	0.069	**0.009**	0.11 (0.01-0.20)	0.226	0.051	**0.025**	0.09 (-0.01-0.19)	0.178	0.032	0.072	0.08 (-0.01-0.16)	0.175	0.030	0.078
Office SBP at follow-up (mmHg)		0.01 (-0.08-0.10)	0.018	0.000	0.833	0.001 (-0.075-0.077)	0.002	0.000	0.977	0.01 (-0.08-0.10)	0.019	0.000	0.817	0.002 (-0.070-0.073)	0.003	0.000	0.966
Maternal smoking during pregnancy (0 = no, 1 = yes)	Smoking	-5.89 (-12.17-0.41)	-0.154	0.024	0.067	-2.38 (-7.84-3.07)	-0.073	0.005	0.389	-6.97 (-13.26- -0.67)	-0.176	0.031	**0.030**	-3.64 (-8.93-1.65)	-0.110	0.012	0.176

B indicates unstandardized beta; 95% CI, 95% confidence interval; R2, nonadjusted R Square. Significant p-values (<0.05) bolded. BMI indicates body mass index; SBP, systolic blood pressure; DBP, diastolic blood pressure; PP, pulse pressure; HR, heart rate; bpm, beats per minute; SGA, small for gestational age (birth weight <-2SD); premature, birth <37+0 gestational weeks; PWV, pulse wave velocity; gestwk = gestational weeks.

Supplementary Table 7. Results of univariate linear regressions for pre-eclamptic children's ambulatory blood pressure and pulse pressure and pulse wave velocities

		**Nighttime systolic blood pressure**				**Nighttime pulse pressure**				**Carotid-femoral pulse wave velocity**				**Carotid-radial pulse wave velocity**			
		B (95% CI)	Standardized B	R^2	*p*	B (95% CI)	Standardized B	R^2	*p*	B (95% CI)	Standardized B	R^2	*p*	B (95% CI)	Standardized B	R^2	*p*
Maternal age at delivery (years)		0.10 (-0.27-0.47)	0.044	0.002	0.584	-0.13 (-0.43-0.17)	-0.071	0.005	0.385	0.02 (-0.01-0.05)	0.111	0.012	0.144	0.02 (-0.02-0.06)	0.067	0.005	0.380
Maternal pre-pregnancy BMI (kg/m^2)		0.29 (-0.07-0.64)	0.129	0.017	0.110	0.61 (0.34-0.88)	0.342	0.117	**<0.001**	0.01 (-0.01-0.04)	0.065	0.004	0.396	0.01 (-0.03-0.05)	0.036	0.001	0.640
Primiparous (0 = yes, 1 = no)		4.71 (0.71-8.72)	0.185	0.034	**0.021**	3.84 (0.56-7.11)	0.185	0.034	**0.022**	-0.08 (-0.36-0.21)	-0.041	0.002	0.593	0.05 (-0.37-0.47)	0.018	0.000	0.814
Gestational weeks at delivery		-0.56 (-1.05- -0.06)	-0.177	0.031	**0.028**	-0.47 (-0.87- -0.07)	-0.185	0.034	**0.022**	0.03 (-0.01-0.07)	0.125	0.016	0.100	-0.01 (-0.07-0.04)	-0.033	0.001	0.666
Premature (0 = no, 1 = yes)		5.17 (1.62-8.72)	0.227	0.052	**0.005**	4.78 (1.91-7.64)	0.259	0.067	**0.001**	-0.21 (-0.46-0.05)	-0.121	0.015	0.112	-0.004 (-0.387-0.378)	-0.002	0.000	0.982
Birth height (cm)	Pregnancy	-0.24 (-0.61-0.13)	-0.102	0.010	0.210	-0.24 (-0.54-0.06)	-0.127	0.016	0.118	0.03 (0.01-0.06)	0.182	0.033	**0.017**	-0.001 (-0.043-0.041)	-0.003	0.000	0.971
Birth height z-score		-0.13 (-1.43-1.17)	-0.016	0.000	0.842	-0.22 (-1.27-0.83)	-0.034	0.001	0.680	0.08 (-0.01-0.17)	0.128	0.016	0.093	-0.01 (-0.15-0.12)	-0.016	0.000	0.840
Birth weight (g)		-0.002 (-0.004-0)	-0.195	0.038	**0.015**	-0.001 (-0.003-0)	-0.149	0.022	0.065	0 (0-0)	0.166	0.028	**0.029**	0 (0-0)	-0.061	0.004	0.424
Birth weight z-score		-1.34 (-2.63- -0.04)	-0.163	0.026	**0.044**	-0.44 (-1.51-0.62)	-0.067	0.004	0.413	0.09 (-0.01-0.18)	0.135	0.018	0.076	-0.11 (-0.25-0.04)	-0.112	0.013	0.143
SGA (0 = no, 1 = yes)		5.28 (0.64-9.91)	0.180	0.032	**0.026**	4.12 (0.37-7.87)	0.174	0.030	**0.032**	0.02 (-0.30-0.34)	0.010	0.000	0.897	0.49 (0.02-0.95)	0.157	0.025	**0.040**
Birth head circumference (cm)		-0.50 (-1.02-0.03)	-0.150	0.023	0.066	-0.41 (-0.84-0.01)	-0.156	0.024	0.057	0.04 (0.01-0.08)	0.175	0.031	**0.022**	-0.02 (-0.08-0.04)	-0.049	0.002	0.525
Birth head circumference z-score		-1.26 (-2.59-0.07)	-0.152	0.023	0.062	-0.85 (-1.93-0.23)	-0.127	0.016	0.121	0.07 (-0.03-0.16)	0.106	0.011	0.167	-0.12 (-0.26-0.02)	-0.132	0.018	0.087
Sex (0 = female, 1 = male)	Sex	2.08 (-1.34-5.49)	0.097	0.009	0.232	0.96 (-1.83-3.74)	0.055	0.003	0.498	0.17 (-0.07-0.41)	0.107	0.011	0.161	0.30 (-0.06-0.66)	0.124	0.015	0.106
Age (years)	Age	0.41 (-1.12-1.93)	0.043	0.002	0.599	1.34 (0.11-2.57)	0.173	0.030	**0.032**	0.21 (0.10-0.32)	0.285	0.081	**<0.001**	0.12 (-0.05-0.28)	0.106	0.011	0.168
Body height (cm)		0.11 (-0.06-0.27)	0.103	0.011	0.205	0.22 (0.09-0.35)	0.266	0.071	**<0.001**	0.03 (0.02-0.04)	0.343	0.118	**<0.001**	0.01 (-0.01-0.03)	0.097	0.009	0.204
Body weight (kg)		0.23 (0.09-0.36)	0.260	0.068	**0.001**	0.30 (0.20-0.40)	0.426	0.181	**<0.001**	0.02 (0.01-0.03)	0.234	0.055	**0.002**	-0.003 (-0.019-0.021)	-0.031	0.001	0.690
Body surface area (m^2)		11.30 (4.05-18.55)	0.242	0.059	**0.002**	15.62 (10.13-21.12)	0.416	0.173	**<0.001**	0.94 (0.41-1.48)	0.257	0.066	**<0.001**	-0.08 (-0.90-0.75)	-0.014	0.000	0.855
Lean body mass (kg)	Anthropometrics	0.29 (0.07-0.52)	0.206	0.042	**0.011**	0.43 (0.26-0.60)	0.374	0.140	**<0.001**	0.04 (0.02-0.05)	0.333	0.111	**<0.001**	0.01 (-0.02-0.04)	0.055	0.003	0.474
Skeletal muscle mass (kg)		0.50 (0.12-0.88)	0.209	0.044	**0.010**	0.72 (0.43-1.01)	0.373	0.139	**<0.001**	0.06 (0.04-0.09)	0.333	0.111	**<0.001**	0.02 (-0.03-0.06)	0.059	0.004	0.440
Height z-score		1.00 (-0.47-2.47)	0.109	0.012	0.180	1.54 (0.37-2.72)	0.206	0.043	**0.011**	0.14 (0.04-0.25)	0.197	0.039	**0.009**	0.03 (-0.13-0.20)	0.029	0.001	0.710
Weight z-score (height)		3.03 (1.55-4.51)	0.312	0.098	**<0.001**	3.24 (2.08-4.39)	0.412	0.170	**<0.001**	-0.02 (-0.14-0.09)	-0.033	0.001	0.670	-0.22 (-0.38- -0.05)	-0.197	0.039	**0.010**
Weight z-score (age)		2.93 (1.44-4.43)	0.299	0.090	**<0.001**	3.47 (2.32-4.62)	0.436	0.190	**<0.001**	0.08 (-0.03-0.20)	0.105	0.011	0.168	-0.15 (-0.32-0.03)	-0.127	0.016	0.096
Waist circumference (cm)		0.34 (0.19-0.49)	0.346	0.120	**<0.001**	0.37 (0.26-0.48)	0.463	0.214	**<0.001**	0.012 (0-0.023)	0.149	0.022	0.050	-0.01 (-0.02-0.01)	-0.062	0.004	0.421
Waist-hip ratio (no unit)		53.04 (27.05-79.03)	0.311	0.097	**<0.001**	36.88 (15.57-58.18)	0.268	0.072	**<0.001**	0.15 (-1.89-2.19)	0.011	0.000	0.884	0.03 (-2.99-3.06)	0.002	0.000	0.984
BMI (kg/m^2)	Adiposity	0.97 (0.51-1.42)	0.322	0.104	**<0.001**	1.08 (0.74-1.43)	0.447	0.200	**<0.001**	0.02 (-0.02-0.05)	0.079	0.006	0.298	-0.048 (-0.101-0.004)	-0.139	0.019	0.070
BMI z-score		3.21 (1.73-4.68)	0.328	0.108	**<0.001**	3.60 (2.46-4.73)	0.454	0.206	**<0.001**	0.02 (-0.10-0.13)	0.025	0.001	0.745	-0.19 (-0.36- -0.03)	-0.172	0.030	**0.024**
Fat mass (kg)		0.45 (0.20-0.70)	0.278	0.077	**<0.001**	0.53 (0.34-0.72)	0.410	0.168	**<0.001**	0.01 (-0.01-0.03)	0.063	0.004	0.409	-0.02 (-0.05-0.01)	-0.117	0.014	0.129
Fat percentage (%)		0.30 (0.11-0.48)	0.247	0.061	**0.002**	0.36 (0.21-0.50)	0.365	0.123	**<0.001**	-0.01 (-0.02-0.01)	-0.054	0.003	0.484	-0.03 (-0.05- -0.01)	-0.187	0.035	**0.014**
SBP (mmHg)		0.49 (0.33-0.65)	0.447	0.199	**<0.001**	0.39 (0.26-0.52)	0.443	0.196	**<0.001**	0.03 (0.01-0.04)	0.282	0.079	**<0.001**	0.022 (0.002-0.041)	0.168	0.028	**0.028**
DBP (mmHg)		0.14 (-0.15-0.43)	0.077	0.006	0.340	0.004 (-0.233-0.240)	0.002	0.000	0.976	0.05 (0.03-0.07)	0.351	0.123	**<0.001**	0.06 (0.03-0.09)	0.303	0.092	**<0.001**
PP (mmHg)	Office BP	0.44 (0.24-0.64)	0.331	0.110	**<0.001**	0.46 (0.30-0.62)	0.419	0.176	**<0.001**	0.01 (-0.01-0.02)	0.054	0.003	0.482	-0.01 (-0.03-0.02)	-0.050	0.002	0.518
HR (bpm)		0.14 (-0.03-0.30)	0.129	0.017	0.116	0.03 (-0.11-0.17)	0.036	0.001	0.666	0.02 (0.01-0.03)	0.251	0.063	**<0.001**	0.018 (0.001-0.036)	0.157	0.025	**0.042**
SBP z-score		5.19 (3.21-7.17)	0.390	0.152	**<0.001**	3.67 (2.03-5.31)	0.341	0.117	**<0.001**	0.12 (-0.03-0.27)	0.125	0.016	0.103	0.18 (-0.05-0.40)	0.118	0.014	0.128
DBP z-score		1.88 (-1.08-4.83)	0.102	0.010	0.212	0.001 (-2.409-2.410)	0.000	0.000	1.000	0.39 (0.19-0.58)	0.288	0.083	**<0.001**	0.62 (0.32-0.91)	0.304	0.093	**<0.001**
Carotid-femoral PWV (m/s)		1.13 (-0.97-3.50)	0.092	0.009	0.265	0.82 (-1.03-2.66)	0.073	0.005	0.384	-	-	-	-	-	-	-	-
Carotid-radial PWV (m/s)		0.86 (-0.54-2.26)	0.101	0.010	0.225	0.29 (-0.87-1.46)	0.042	0.002	0.618	-	-	-	-	-	-	-	-
Central SBP (mmHg)	PWV	0.35 (0.22-0.47)	0.414	0.171	**<0.001**	0.33 (0.23-0.42)	0.474	0.225	**<0.001**	-	-	-	-	-	-	-	-
Central DBP (mmHg)		0.22 (-0.07-0.52)	0.124	0.015	0.133	0.03 (-0.21-0.27)	0.021	0.000	0.800	-	-	-	-	-	-	-	-
Central PP (mmHg)		0.33 (0.19-0.46)	0.371	0.138	**<0.001**	0.35 (0.24-0.45)	0.485	0.235	**<0.001**	-	-	-	-	-	-	-	-
SBP at first antenatal visit (mmHg)		0.11 (-0.05-0.26)	0.111	0.012	0.175	0.128 (0.003-0.254)	0.164	0.027	**0.045**	0.010 (-0.001-0.020)	0.135	0.018	0.080	0.01 (-0.01-0.02)	0.055	0.003	0.480
Highest SBP before 20 gestwk (mmHg)	Maternal BP	0.14 (-0.02-0.30)	0.169	0.029	0.087	0.18 (0.04-0.31)	0.251	0.063	**0.010**	0.01 (-0.01-0.02)	0.108	0.012	0.249	0.01 (-0.01-0.03)	0.085	0.007	0.368
Highest SBP from 20 gestwk to childbirth (mmHg)		0.17 (0.04-0.29)	0.247	0.061	**0.011**	0.13 (0.02-0.24)	0.232	0.054	**0.019**	0.01 (-0.01-0.02)	0.087	0.008	0.355	0.01 (-0.01-0.02)	0.088	0.008	0.353
Office SBP at follow-up (mmHg)		0.03 (-0.07-0.14)	0.050	0.002	0.541	0.05 (-0.03-0.14)	0.101	0.010	0.214	0.011 (0.004-0.018)	0.227	0.051	**0.003**	0.001 (-0.010-0.012)	0.008	0.000	0.918
Maternal smoking during pregnancy (0 = no, 1 = yes)	Smoking	-3.48 (-11.17-4.21)	-0.073	0.005	0.372	1.45 (-4.77-7.67)	0.038	0.001	0.645	-0.01 (-0.58-0.57)	-0.001	0.000	0.986	0.04 (-0.82-0.90)	0.008	0.000	0.922

B indicates unstandardized beta; 95% CI, 95% confidence interval; R2, nonadjusted R Square. Significant p-values (<0.05) bolded. BMI indicates body mass index; SBP, systolic blood pressure; DBP, diastolic blood pressure; PP, pulse pressure; HR, heart rate; bpm, beats per minute; SGA, small for gestational age (birth weight <-2SD); premature, birth <37+0 gestational weeks; PWV, pulse wave velocity; gestwk = gestational weeks.

Supplementary Table 8. Adjusted mean differences for pre-eclamptic versus non-pre-eclamptic children

Outcome	Unadjusted mean difference (95% CI)	Adjustment variable	PE Adjusted mean (SE)	non-PE Adjusted mean (SE)	Adjusted mean difference (95% CI)	P-value
**24-hour SBP (mmHg)**	2.9 (0.4-5.3)	Maternal SBP at first antenatal visit (mmHg)	122.1 (0.7)	120.4 (1.1)	1.7 (-0.9-4.4)	0.190
		Maternal parity (0 = primiparous, 1 = multiparous)	122.6 (0.7)	119.4 (1.0)	3.2 (0.7-5.6)	**0.013**
		Prematurity (0 = no, 1 = yes)	122.1 (0.7)	120.52 (1.1)	1.6 (-1.0-4.1)	0.225
		Gestational weeks at delivery	122.1 (0.7)	120.6 (1.1)	1.4 (-1.3-4.1)	0.300
		Child birth weight (g)	122.0 (0.7)	120.7 (1.1)	1.4 (-1.3-4.1)	0.319
		Child birth weight z-score	122.3 (0.7)	120.0 (1.1)	2.3 (-0.2-4.9)	0.068
		Child waist-hip ratio at follow-up (no unit)	122.4 (0.7)	119.7 (1.0)	2.7 (0.3-5.1)	**0.030**
		Child BMI z-score at follow-up	122.5 (0.7)	119.6 (1.0)	2.9 (0.5-5.3)	**0.020**
		Child weight z-score (height) at follow-up	122.5 (0.7)	119.6 (1.0)	2.9 (0.4-5.3)	**0.021**
**24-hour PP (mmHg)**	3.7 (1.9-5.4)	Maternal SBP at first antenatal visit (mmHg)	51.9 (0.6)	48.8 (0.9)	3.1 (0.9-5.3)	**0.007**
		Maternal parity (0 = primiparous, 1 = multiparous)	52.2 (0.6)	48.1 (0.9)	4.1 (2.1-6.1)	**<0.001**
		Maternal pre-pregnancy BMI (kg/m^2)	51.9 (0.6)	48.8 (0.9)	3.1 (1.0-5.1)	**0.004**
		Prematurity (0 = no, 1 = yes)	51.7 (0.6)	49.4 (0.9)	2.3 (0.2-4.4)	**0.034**
		Gestational weeks at delivery	51.7 (0.6)	49.3 (0.9)	2.5 (0.2-4.7)	**0.033**
		Child birth weight (g)	51.7 (0.6)	49.2 (0.9)	2.6 (0.3-4.8)	**0.027**
		Child lean body mass at follow-up (kg)	52.0 (0.6)	48.7 (0.8)	3.3 (1.3-5.3)	**0.001**
		Child waist-hip ratio at follow-up (no unit)	52.0 (0.6)	48.5 (0.9)	3.6 (1.5-5.6)	**<0.001**
		Child BMI z-score at follow-up	52.1 (0.5)	48.4 (0.8)	3.7 (1.8-5.6)	**<0.001**
		Child weight z-score (height) at follow-up	52.1 (0.6)	48.4 (0.8)	3.7 (1.7-5.7)	**<0.001**
		Child fat mass at follow-up (kg)	52.0 (0.6)	48.7 (0.8)	3.3 (1.3-5.4)	**0.001**
		Child body fat percentage	52.1 (0.6)	48.5 (0.9)	3.6 (1.5-5.6)	**<0.001**
**CF-PWV (m/s)**	0.20 (0-0.41)	Child birth weight (g)	5.36 (0.06)	5.04 (0.09)	0.32 (0.09-0.55)	**0.006**
		Child age at follow-up (years)	5.30 (0.06)	5.16 (0.08)	0.14 (-0.06-0.34)	0.179
		Child body height at follow-up (cm)	5.31 (0.06)	5.14 (0.08)	0.17 (-0.03-0.37)	0.087
		Child body weight at follow-up (kg)	5.32 (0.06)	5.13 (0.09)	0.19 (-0.01-0.39)	0.063
		Child office SBP at follow-up visit (mmHg)	5.29 (0.06)	5.19 (0.09)	0.09 (-0.11-0.30)	0.372
		Child office HR at follow-up visit (bpm)	5.33 (0.06)	5.12 (0.08)	0.21 (0.01-0.42)	**0.036**
		Maternal office SBP at follow-up	5.30 (0.06)	5.18 (0.09)	0.12 (-0.09-0.32)	0.265

PE indicates pre-eclampsia; non-PE, control group; SE, standard error; CI, confidence interval; SBP, systolic blood pressure; PP, pulse pressure;
CF-PWV, carotid-femoral pulse wave velocity; gestwk, gestational weeks; BMI, body mass index; HR, heart rate; bpm, beats per minute; prematurity, birth <37+0 gestational weeks.
Significant p-values (<0.05) bolded.

Supplementary Table 9. Multivariable linear regression models for children's 24-hour SBP, 24-hour PP and carotid-femoral PWV						
**Dependent**	**Predictor**	**Unstandardized β (95% CI)**	**Standardized β**	**P-value**	**Adjusted R2**	**Model p-value**
**24-hour SBP**						
**Model 1**	Constant	119.64 (117.58-121.69)			0.020	**0.024**
	Pre-eclampsia (0 = no, 1 = yes)	2.85 (0.39-5.31)	0.157	**0.024**		
**Model 2**	Constant	119.78 (117.75-121.80)			0.051	**0.002**
	Pre-eclampsia (0 = no, 1 = yes)	2.88 (0.46-5.31)	0.159	**0.020**		
	Child BMI z-score at follow-up	1.53 (0.45-2.62)	0.189	**0.006**		
**Model 3**	Constant	118.97 (116.75-121.18)			0.061	**0.001**
	Pre-eclampsia (0 = no, 1 = yes)	3.16 (0.73-5.60)	0.175	**0.011**		
	Child BMI z-score at follow-up	1.49 (0.41-2.57)	0.184	**0.007**		
	Maternal parity (0 = primiparous, 1 = multiparous)	2.20 (-0.31-4.71)	0.118	0.085		
**Model 4a**	Constant	126.66 (121.24-132.07)			0.098	**<0.001**
	Pre-eclampsia (0 = no, 1 = yes)	1.47 (-1.15-4.10)	0.081	0.270		
	Child BMI z-score at follow-up	1.60 (0.54-2.66)	0.197	**0.003**		
	Maternal parity (0 = primiparous, 1 = multiparous)	2.64 (0.17-5.12)	0.142	**0.037**		
	Child birth weight (g)	-0.002 (-0.004- -0.001)	-0.226	**0.003**		
**Model 4b**	Constant	105.62 (94.22-117.02)			0.077	**<0.001**
	Pre-eclampsia (0 = no, 1 = yes)	2.02 (-0.56-4.59)	0.112	0.124		
	Child BMI z-score at follow-up	1.47 (0.40-2.54)	0.183	**0.007**		
	Maternal parity (0 = primiparous, 1 = multiparous)	2.05 (-0.45-4.55)	0.110	0.107		
	Maternal SBP at first antenatal visit (mmHg)	0.12 (0.02-0.22)	0.171	**0.019**		
**Model 5**	Constant	115.88 (103.36-128.41)			0.126	**<0.001**
	Pre-eclampsia (0 = no, 1 = yes)	0.19 (-2.52-2.90)	0.011	0.890		
	Child BMI z-score at follow-up	1.59 (0.55-2.64)	0.198	**0.003**		
	Maternal parity (0 = primiparous, 1 = multiparous)	2.46 (0.01-4.90)	0.132	**0.049**		
	Child birth weight (g)	-0.003 (-0.004- -0.001)	-0.256	**<0.001**		
	Maternal SBP at first antenatal visit (mmHg)	0.11 (0.01-0.20)	0.154	**0.030**		
**24-hour PP**						
**Model 1**	Constant	48.43 (46.71-50.15)			0.051	**<0.001**
	Pre-eclampsia (0 = no, 1 = yes)	3.65 (1.58-5.71)	0.236	**<0.001**		
**Model 2**	Constant	48.64 (47.02-50.26)			0.159	**<0.001**
	Pre-eclampsia (0 = no, 1 = yes)	3.69 (1.75-5.64)	0.239	**<0.001**		
	Child BMI z-score at follow-up	2.31 (1.44-3.18)	0.334	**<0.001**		

**Model 3**	Constant	47.40 (45.66-49.14)			0.201	**<0.001**
	Pre-eclampsia (0 = no, 1 = yes)	4.13 (2.22-6.04)	0.268	**<0.001**		
	Child BMI z-score at follow-up	2.24 (1.39-3.09)	0.324	**<0.001**		
	Maternal parity (0 = primiparous, 1 = multiparous)	3.39 (1.42-5.37)	0.213	**<0.001**		
**Model 4a**	Constant	53.86 (49.62-58.10)			0.237	**<0.001**
	Pre-eclampsia (0 = no, 1 = yes)	2.71 (0.65-4.76)	0.175	**0.010**		
	Child BMI z-score at follow-up	2.33 (1.50-3.16)	0.337	**<0.001**		
	Maternal parity (0 = primiparous, 1 = multiparous)	3.77 (1.83-5.70)	0.237	**<0.001**		
	Child birth weight (g)	-0.002 (-0.003- -0.001)	-0.223	**0.001**		
**Model 4b**	Constant	40.45 (31.41-49.48)			0.200	**<0.001**
	Pre-eclampsia (0 = no, 1 = yes)	3.51 (1.47-5.55)	0.229	**<0.001**		
	Child BMI z-score at follow-up	2.22 (1.38-3.07)	0.325	**<0.001**		
	Maternal parity (0 = primiparous, 1 = multiparous)	3.27 (1.29-5.25)	0.206	**0.001**		
	Maternal SBP at first antenatal visit (mmHg)	0.06 (-0.02-0.14)	0.105	0.120		
**Model 5**	Constant	49.09 (39.20-58.98)			0.248	**<0.001**
	Pre-eclampsia (0 = no, 1 = yes)	1.97 (-0.17-4.11)	0.129	0.071		
	Child BMI z-score at follow-up	2.33 (1.51-3.15)	0.341	**<0.001**		
	Maternal parity (0 = primiparous, 1 = multiparous)	3.61 (1.68-5.54)	0.228	**<0.001**		
	Child birth weight (g)	-0.002 (-0.003- -0.001)	-0.253	**<0.001**		
	Maternal SBP at first antenatal visit (mmHg)	0.05 (-0.02-0.13)	0.09	0.181		
**CF-PWV**						
**Model 1**	Constant	5.12 (4.95-5.29)			0.011	**0.049**
	Pre-eclampsia (0 = no, 1 = yes)	0.20 (0-0.41)	0.124	**0.049**		
**Model 2**	Constant	4.62 (4.14-5.10)			0.026	**0.014**
	Pre-eclampsia (0 = no, 1 = yes)	0.32 (0.09-0.55)	0.193	**0.006**		
	Child birth weight (g)	0 (0-0)	0.153	**0.030**		
**Model 3**	Constant	2.46 (1.38-3.55)			0.091	**<0.001**
	Pre-eclampsia (0 = no, 1 = yes)	0.26 (0.04-0.48)	0.157	**0.022**		
	Child birth weight (g)	0 (0-0)	0.164	**0.016**		
	Child age at follow-up (years)	0.19 (0.10-0.27)	0.265	**<0.001**		
**Model 4a**	Constant	0.50 (-0.98-1.97)			0.138	**<0.001**
	Pre-eclampsia (0 = no, 1 = yes)	0.18 (-0.04-0.40)	0.111	0.101		
	Child birth weight (g)	0 (0-0)	0.186	**0.005**		
	Child age at follow-up (years)	0.16 (0.08-0.25)	0.229	**<0.001**		
	Child office SBP at follow-up visit (mmHg)	0.02 (0.01-0.03)	0.233	**<0.001**		

**Model 4b**	Constant	1.42 (0.18-2.66)			0.126	**<0.001**
	Pre-eclampsia (0 = no, 1 = yes)	0.20 (-0.02-0.42)	0.120	0.078		
	Child birth weight (g)	0 (0-0)	0.187	**0.006**		
	Child age at follow-up (years)	0.17 (0.09-0.25)	0.240	**<0.001**		
	Maternal office SBP at follow-up visit (mmHg)	0.009 (0.004-0.015)	0.205	**0.001**		
**Model 5**	Constant	-0.25 (-1.80-1.29)			0.163	**<0.001**
	Pre-eclampsia (0 = no, 1 = yes)	0.13 (-0.09-0.35)	0.082	0.228		
	Child birth weight (g)	0 (0-0)	0.202	**0.002**		
	Child age at follow-up (years)	0.15 (0.07-0.23)	0.211	**<0.001**		
	Child office SBP at follow-up visit (mmHg)	0.02 (0.01-0.03)	0.211	**<0.001**		
	Maternal office SBP at follow-up visit (mmHg)	0.008 (0.003-0.014)	0.181	**0.003**		

Significant results are bolded (p-value <0.05). CI indicates confidence interval; SBP, systolic blood pressure; BMI, body mass index; PP, pulse pressure; CF-PWV, carotid-femoral pulse wave velocity.

Supplementary Table 10. Primi- versus multiparous PE mothers	Primiparous PE	Multiparous PE	P-value primi versus multi	Mean difference (95% CI)
**Maternal pregnancy data**	*N = 139*	*N = 43*		
Maternal age at delivery (years)	30.6 (4.4)	33.3 (5.1)	**<0.001**	-2.7 (-4.3- -1.1)
Pre-pregnancy BMI (kg/m^2)	24.5 (4.7)	25.5 (5.1)	0.227	-1.0 (-2.6-0.6)
PE in previous pregnancy, n (%)	0 (0)	16 (37.2)	**<0.001**	-
Maternal SBP at first antenatal visit (mmHg)	122.3 (11.7)	123.9 (11.1)	0.440	-1.6 (-5.6-2.5)
Maternal DBP at first antenatal visit (mmHg)	76.5 (8.2)	79.5 (10.0)	**0.049**	-3.0 (-6.1-0)
Highest SBP during pregnancy (mmHg)	173.7 (16.2)	171.9 (16.6)	0.612	1.8 (-5.1-8.6)
Highest DBP during pregnancy (mmHg)	109.5 (8.5)	108.8 (9.3)	0.701	0.7 (-2.9-4.4)
Smoking during pregnancy, n (%)	7 (5.1)	2 (4.7)	1.000	-
Chronic hypertension, n (%)†	15 (10.8)	8 (18.6)	0.193	-
HELLP syndrome, n (%)	13 (9.4)	1 (2.3)	0.193	-
Eclampsia, n (%)	1 (0.7)	0 (0)	1.000	-
Placental insufficiency, n (%)‡	12 (8.6)	2 (4.7)	0.525	-
Early-onset PE by diagnosis, n (%)	33 (23.7)	13 (30.2)	0.424	-
**Perinatal data**				
Premature, n (%)	46 (33.1)	14 (32.6)	1.000	-
Gestational weeks at delivery	37.9 (3.6)*	38.4 (3.7)*	0.596	-0.3 (-1.0-0.6)
SGA, n (%)	27 (19.4)	5 (11.6)	0.265	-
Child birth weight (g)	2723 (829)	2917 (875)	0.188	-194 (-483-96)
Child birth weight z-score	-0.78 (1.17)	-0.69 (1.55)	0.681	-0.09 (-0.53-0.35)
Child birth height z-score	-0.60 (1.16)	-0.41 (1.70)	0.410	-0.19 (-0.64-0.26)

Data is presented as mean (SD) unless stated otherwise, significant p-values (<0.05) bolded. Independent Samples t Test for normally distributed numerical data, Mann-Whitney U Test for non-normal distribution and Pearson Chi-Square test or Fisher's Exact Test for categorical data. PE indicates pre-eclampsia; BMI, body mass index; SD, standard deviation; IQR, interquartile range; CI, confidence interval; SBP, systolic blood pressure;DBP, diastolic blood pressure; SGA, small for gestational age (birth weight <-2SD); HELLP syndrome, hemolysis, trombocytopenia and elevated liver enzymes; premature, birth <37+0 gestational weeks.

*Median (IQR), median difference (95% CI)

†Systolic blood pressure ≥ 140 mmHg and/or diastolic blood pressure ≥ 90 mmHg detected before 20 weeks of gestation

‡Pulsatility index > +2SD or resistance index > +2SD in umbilical arterial Doppler assessment
